# HIF-1 Modulates Dietary Restriction-Mediated Lifespan Extension via IRE-1 in *Caenorhabditis elegans*

**DOI:** 10.1371/journal.pgen.1000486

**Published:** 2009-05-22

**Authors:** Di Chen, Emma Lynn Thomas, Pankaj Kapahi

**Affiliations:** Buck Institute for Age Research, Novato, California, United States of America; Huntsman Cancer Institute, United States of America

## Abstract

Dietary restriction (DR) extends lifespan in various species and also slows the onset of age-related diseases. Previous studies from flies and yeast have demonstrated that the target of rapamycin (TOR) pathway is essential for longevity phenotypes resulting from DR. TOR is a conserved protein kinase that regulates growth and metabolism in response to nutrients and growth factors. While some of the downstream targets of TOR have been implicated in regulating lifespan, it is still unclear whether additional targets of this pathway also modulate lifespan. It has been shown that the hypoxia inducible factor-1 (HIF-1) is one of the targets of the TOR pathway in mammalian cells. HIF-1 is a transcription factor complex that plays key roles in oxygen homeostasis, tumor formation, glucose metabolism, cell survival, and inflammatory response. Here, we describe a novel role for HIF-1 in modulating lifespan extension by DR in *Caenorhabditis elegans*. We find that HIF-1 deficiency results in extended lifespan, which overlaps with that by inhibition of the RSKS-1/S6 kinase, a key component of the TOR pathway. Using a modified DR method based on variation of bacterial food concentrations on solid agar plates, we find that HIF-1 modulates longevity in a nutrient-dependent manner. The *hif-1* loss-of-function mutant extends lifespan under rich nutrient conditions but fails to show lifespan extension under DR. Conversely, a mutation in *egl-9*, which increases HIF-1 activity, diminishes the lifespan extension under DR. This deficiency is rescued by tissue-specific expression of *egl-9* in specific neurons and muscles. Increased lifespan by *hif-1* or DR is dependent on the endoplasmic reticulum (ER) stress regulator inositol-requiring protein-1 (IRE-1) and is associated with lower levels of ER stress. Therefore, our results demonstrate a tissue-specific role for HIF-1 in the lifespan extension by DR involving the IRE-1 ER stress pathway.

## Author Summary

Dietary restriction (DR) is one of the most robust environmental manipulations that extend lifespan in various species. DR has also been shown to slow the onset of a number of age-related diseases. Studies in model organisms like *C. elegans* can be used to uncover biological mechanisms that determine the beneficial effects of DR. Previous studies suggest that the nutrient-sensing target of rapamycin (TOR) pathway is required for DR-mediated lifespan extension. However, the downstream mechanisms by which TOR modulates lifespan remain unclear. In mammalian cells, TOR and the downstream S6 kinase (S6K) activate expression of the hypoxia-inducible factor-1 (HIF-1), which is frequently up-regulated in various tumors. Using *C. elegans* as a model system, we characterized novel functions of HIF-1 in aging. We find that inhibition of HIF-1 extends lifespan under rich nutrient conditions, whereas enhanced levels of HIF-1 only allow partial lifespan extension by DR. We also demonstrated that increased lifespan by *hif-1* or DR depends on the endoplasmic reticulum (ER) stress regulator inositol-requiring protein-1 (IRE-1) and is associated with lower levels of ER stress, which is caused by overloading of misfolded/unfolded proteins to ER. Thus, our results support the idea that HIF-1–mediated changes in protein homeostasis play a key role in the lifespan extension by DR.

## Introduction

Dietary restriction (DR) has been shown to extend lifespan in various species. It also slows the onset of a number of age-related diseases in rodents. Conservation of signaling pathways in multiple species and the rapidity with which lifespan studies can be carried out in simple model organisms make them powerful tools to understand aging and age-related diseases in humans. Identification of genes involved in DR response will therefore provide potential targets for treatments of age–associated diseases and the extension of healthful lifespan in humans. The quest to understand the mechanisms of DR-induced lifespan extension has led to intensive studies in the primary genetic model organisms *Saccharomyces cerevisiae*, *Caenorhabditis elegans*, and *Drosophila melanogaster*, in which the robust effects of lifespan extension by DR can be observed [1]–[5]. Evidence from previous studies has identified the TOR pathway as a key mediator of nutrient-modulated lifespan changes in flies [6], yeast [7] and worms [8], although the involvement of TOR in response to DR in *C. elegans* is still controversial [8],[9].

TOR is a conserved protein kinase that plays essential roles in regulating growth and metabolism in response to nutrients and growth factors [10]. TOR interacts with the regulatory associated protein of TOR (raptor) to permit transduction of nutrient signals to downstream cellular processes, including mRNA translation, ribosome synthesis, expression of metabolism-related genes and autophagy [11]. TOR promotes protein synthesis by activating S6K and inhibiting eukaryotic translation initiation factor 4E-binding protein (4E-BP). Recent studies have shown that regulation of mRNA translation plays critical roles in lifespan determination in multiple species [9], [12]–[19]. In *C. elegans*, mutations in the TOR ortholog *let-363* lead to developmental arrest at the third larval stage and intestinal atrophy [20]. Inhibition of *let-363* by RNAi extends lifespan [8],[12],[21]. The larval arrest phenotype was also observed from homozygous mutants of *daf-15*, which encodes the *C. elegans* ortholog of raptor. Heterozygous mutants of *daf-15* have increased lifespan [22]. Inactivation of *rsks-1*, which encodes the *C. elegans* ortholog of S6K [23], significantly extends lifespan [12],[13]. Although it is well established that the *C. elegans* TOR pathway modulates lifespan, it is still unclear whether TOR affects DR-mediated lifespan extension. There have been two reports with opposite results on whether inhibition of *let-363* by RNAi further extends lifespan of the *eat-2* mutant, which serves as a genetic mimic of DR [8],[9].

Multiple studies in mammalian and *Drosophila* cells have implicated the transcription factor HIF-1 as a target of the TOR pathway [24]–[27]. HIF-1 is regulated at the mRNA translation level, and enhanced levels of HIF-1 are associated with increased TOR and S6K activities under both normoxic and hypoxic conditions [24],[26],[28],[29]. HIF-1 is a heterodimeric transcriptional complex that contains HIF-1α and HIF-1β. It plays essential roles in oxygen homeostasis [30] and is also regulated by other physiological stimuli like heat acclimation [31], acidosis [32], nitric oxide [33], inflammation [34] and oxidative stress [35],[36]. Under normoxia, specific proline residues of HIF-1α are hydroxylated by the PH superfamily of dioxygenase encoded by *egl-9* in *C. elegans*. Hydroxylated HIF-1α is subject to von Hippel Lindau (pVHL)-mediated proteasome degradation. Under hypoxia, the hydroxylation modification declines and HIF-1α is stabilized for its transcriptional activities [37]. HIF-1 helps cells adapt to low-oxygen stress by regulating angiogenesis, glycolysis, and cell survival [30]. HIF-1 overexpression is frequently detected in solid tumors due to intratumoral hypoxia and genetic mutations, and inhibition of HIF-1 can prevent tumor growth [38],[39]. In *C. elegans*, *hif-1* encodes the HIF-1α ortholog, which has been connected to multiple biological processes, including hypoxia response [37],[40], adaption to heat stress [31], oxygen preference [41],[42], and neuronal migration [43].

Despite the well-characterized links between HIF-1 and cancer, metabolism, and cell survival, it is not known whether HIF-1 is directly involved in organismal aging. In this study, we investigated the role of HIF-1 in lifespan determination in *C. elegans*. We demonstrate that HIF-1 functions downstream of S6K to modulate DR-dependent lifespan extension in specific neurons and muscles via the IRE-1 ER stress pathway.

## Results

### HIF-1 Functions Downstream of S6K to Modulate *C. elegans* Lifespan

**Figure 1:**
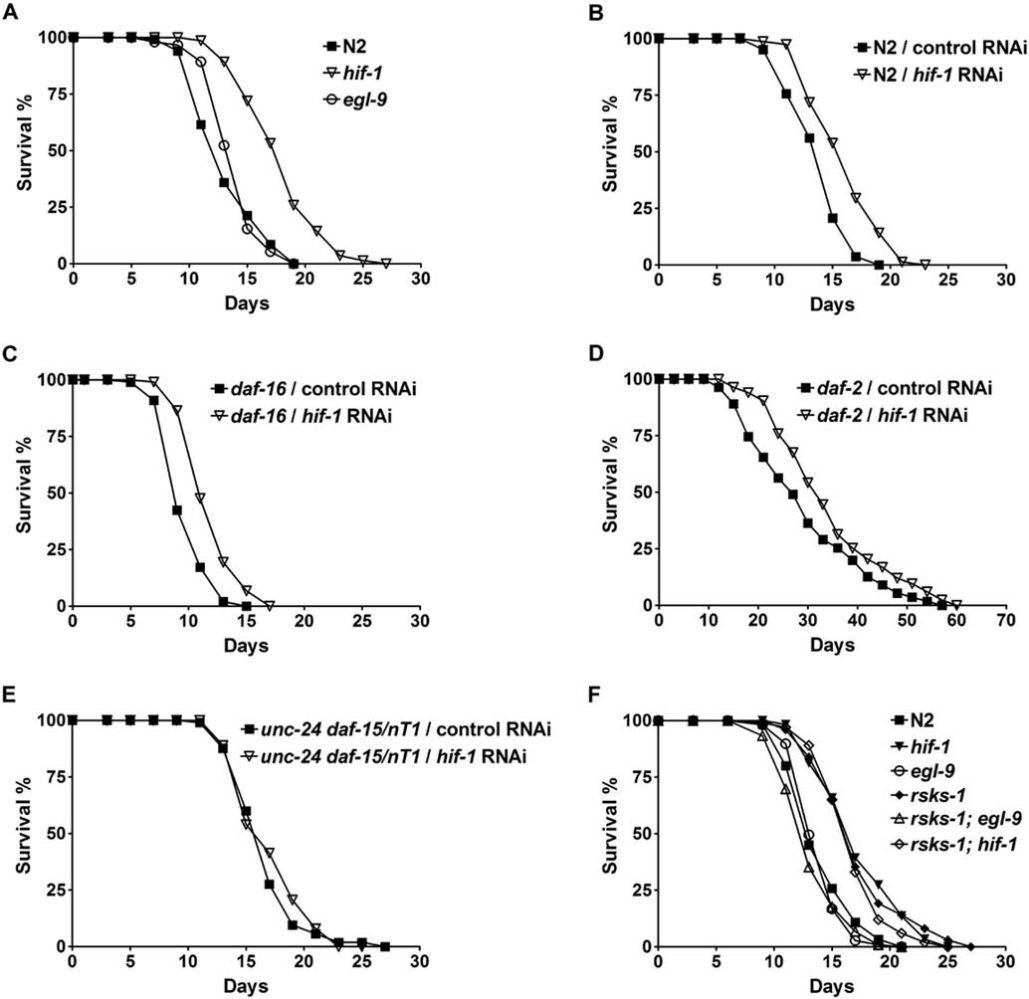
HIF-1 functions downstream of S6K to modulate *C. elegans* lifespan. (A) The *hif-1(ia04)* deletion mutant extends lifespan, whereas the *egl-*9*(sa307)* deletion mutant does not affect lifespan significantly under standard lab culture conditions. (B)–(D) Inhibition of *hif-1* by RNAi extends lifespan of (B) wild-type N2, (C) *daf-16(mgDf47)*, and (D) *daf-2(e1370)* animals. (E) Inhibition of *hif-1* by RNAi does not further extend lifespan of an *unc-24(e138) daf-15(m634)/nT1* heterozygous mutant. (F) *egl-9* suppresses lifespan extension by the *rsks-1(ok1255)* mutation, and *hif-1* does not further increase lifespan of *rsks-1*. Each of the lifespan experiments was performed twice with consistent results. Quantitative data and statistical analyses for the experiments shown here and the repeated experiments are included in Table 1 and Table S1, respectively.

To characterize the role of HIF-1 in organismal aging, we examined lifespan phenotypes of *hif-1* and *egl-9* mutants. The *egl-9* mutant has significantly increased HIF-1 protein levels and transcriptional activities [37],[44], thus serving as a HIF-1 gain-of-function mutant. A deletion mutant of *hif-1* extended lifespan by 24%, whereas the *egl-9* deletion mutant did not affect lifespan significantly under standard lab culture conditions (Figure 1A; Table 1 and S1). Inhibition of *hif-1* by RNAi also extended adult lifespan (Figure 1B; Table 1 and S1). Thus, HIF-1 is a novel lifespan determinant in *C. elegans*.

**Table 1:** HIF-1 functions in the TOR-S6K pathway to modulate *C. elegans* lifespan.

Genotype	RNAi	Mean lifespan a	Percent of control b	n c	*p*-value vs. control d
N2	− e	13.8±1.0	−	117	−
*hif-1(ia04)*	− e	17.1±0.8	124%	139	<0.0001
*egl-9(sa307)*	− e	14.5±1.4	105%	149	0.0905
N2	control	13.6±0.6	−	82	−
N2	*hif-1*	16.1±0.4	118%	78	<0.0001
*daf-16(mgDf47)*	control	10.5±0.6	−	99	−
*daf-16(mgDf47)*	*hif-1*	12.4±0.2	118%	103	<0.0001
*daf-2(e1370)*	control	29.0±0.3	−	55	−
*daf-2(e1370)*	*hif-1*	35.0±0.8	121%	83	0.0191
*unc-24(e138) daf-15(m634)/nT1*	control	16.6±0.4	−	105	−
*unc-24(e138) daf-15(m634)/nT1*	*hif-1*	16.6±1.1	100%	103	0.3407
N2	− e	14.0±0.6	−	120	−
*hif-1(ia04)*	− e	17.3±0.5	124%	117	<0.0001
*egl-9(sa307)*	− e	14.5±0.4	104%	107	0.4490
*rsks-1(ok1255)*	− e	17.1±1.3	122%	99	<0.0001
*rsks-1(ok1255); hif-1(ia04)* f	− e	17.0±0.3	121%	100	<0.0001
*rsks-1(ok1255); egl-9(sa307)* g	− e	14.2±0.7	101%	119	0.0311

The lifespan experiments were performed twice with consistent results. Data for representative experiments are shown in this table, and data for repeated experiments are shown in Table S1.

a average lifespan±standard deviation in days.

b percentages were calculated using the mean lifespan.

c numbers of animals scored.

d *p*-values were calculated for log-rank tests.

e animals were treated with *E. coli* OP50 food under standard lab conditions.

f log-rank test: *rsks-1* vs. *rsks-1; hif-1*, *p* = 0.2142.

g log-rank test: *rsks-1* vs. *rsks-1; egl-9*, *p*<0.0001.

In order to characterize the mechanism of lifespan extension by *hif-1* deficiency, we performed genetic epistasis experiments to test interactions between *hif-1* and other known longevity pathways. We tested mutations from the insulin/insulin-like growth facor-1 (IIS) pathway, which is a conserved pathway that modulates lifespan in nematodes, flies and mammals [3]. Mutations in the DAF-2/IGF-1 receptor double *C. elegans* lifespan [45],[46], and this lifespan extension is suppressed by mutations in the downstream DAF-16/FOXO transcription factor [47],[48]. Lifespan extension by *hif-1* did not require DAF-16 (Figure 1C; Table 1 and S1). Inhibition of *hif-1* by RNAi extended lifespan of N2 and a *daf-16* null mutant to similar levels (18% lifespan extension, *p*<0.0001). Consistently, *hif-1* RNAi further extended the lifespan of a *daf-2* mutant (Figure 1D; Table 1 and S1). These results suggest that HIF-1 might modulate lifespan through mechanisms that are distinct from those used by the IIS pathway.

Next we tested the interaction between HIF-1 and the TOR-S6K pathway since previous studies from mammalian cells have shown that S6K promotes *hif-1* expression [24],[26],[28],[29]. We found that inhibition of *hif-1* by RNAi did not further extend lifespan of a *daf-15* heterozygous mutant (Figure 1E; Table 1 and S1). The lifespan extension by a deletion mutant of *rsks-1* was fully suppressed by *egl-9*, and *hif-1* did not further extend *rsks-1* lifespan (Figure 1F; Table 1 and S1). Taken together, these experiments suggest that lifespan extension by inhibition of *rsks-1* and *hif-1* takes place through overlapping mechanisms. We hypothesize that HIF-1 acts downstream of the nutrient-responsive TOR-S6K pathway to determine lifespan in *C. elegans*.

### HIF-1 Modulates Lifespan Extension by DR

**Figure 2:**
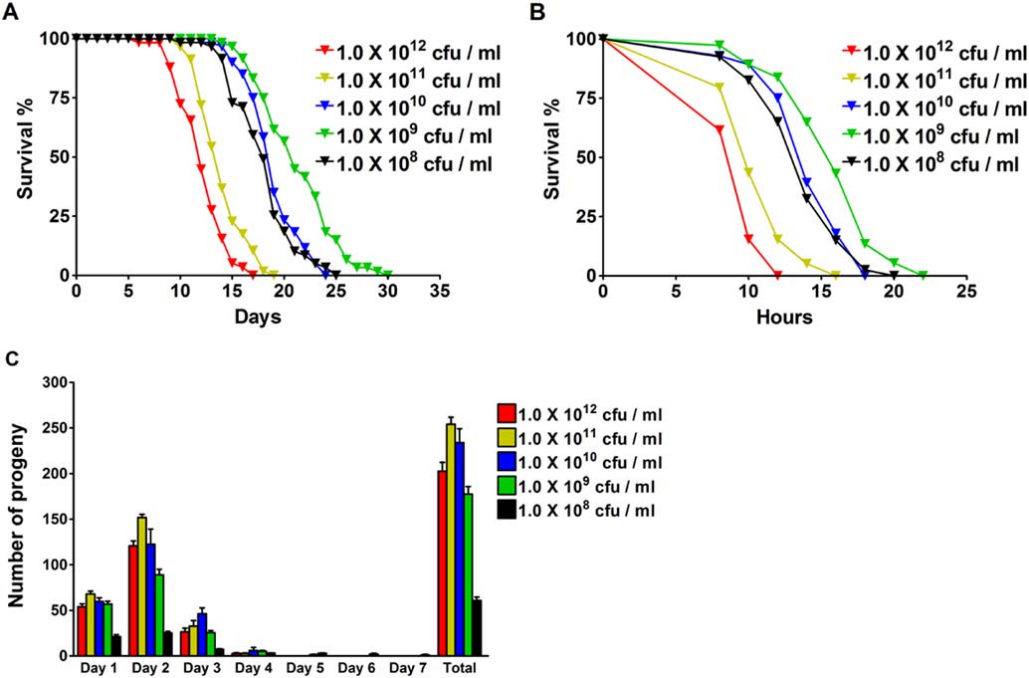
DR extends lifespan, increases stress resistance and decreases fecundity in *C. elegans*. (A) Survival curves of N2 animals fed with *E. coli* at different concentrations (1.0×10^8^ to 1.0×10^12^ cfu/ml) during adulthood. Mean lifespan was 12.2 days for 1.0×10^12^ cfu/ml, 14.0 days for 1.0×10^11^ cfu/ml, 18.9 days for 1.0×10^10^ cfu/ml, 21.2 days for 1.0×10^9^ cfu/ml, and 18.0 days for 1.0×10^8^ cfu/ml. (B) Heat stress resistance of adults after maintenance under different nutrient conditions. Mean survival was 9.5 hours for 1.0×10^12^ cfu/ml, 10.9 hours for 1.0×10^11^ cfu/ml, 14.3 hours for 1.0×10^10^ cfu/ml, 15.9 hours for 1.0×10^9^ cfu/ml, and 13.8 hours for 1.0×10^8^ cfu/ml. (C) Egg production after maintenance under different nutrient conditions. Average brood sizes were 203±37 for 1.0×10^12^ cfu/ml, 254±30 for 1.0×10^11^ cfu/ml, 234±60 for 1.0×10^10^ cfu/ml, 178±32 for 1.0×10^9^ cfu/ml, and 61±15 for 1.0×10^8^ cfu/ml. Fifteen animals were scored for each food concentration.

Next we investigated whether HIF-1 is involved in nutrient-mediated lifespan extension using a DR paradigm modified from the previously described solid DR (sDR) method [49]. Lifespan of animals fed with bacterial food at different concentrations (1.0×10^8^–1.0×10^12^ cfu/ml) was examined on solid agar plates. The differences between our DR paradigm and the sDR method include that peptone was excluded and antibiotics were added to prevent bacterial growth, 5-fluorodeoxyuridine (FUdR) was used to prevent progeny from hatching, and differential food treatment was started from Day 1 instead of Day 4 post-reproductive adulthood. We have termed this method as msDR for modified solid DR. As shown in Figure 2A, animals treated with *E. coli* food at 1.0×10^11^ cfu/ml had a lifespan similar to those under standard culture conditions, whereas animals fed with *E. coli* at 1.0×10^9^ cfu/ml showed the most significant lifespan extension (47% lifespan extension compared to 1.0×10^11^ cfu/ml, *p*<0.0001). We also observed that reductions in bacterial concentration led to increased heat stress resistance and decreased fecundity (Figure 2B and 2C), consistent with the phenotypes observed from other model organisms under DR [50],[51]. Based on the tradeoffs between fecundity and optimal lifespan extension and stress resistance, bacterial concentrations at 1.0×10^11^ cfu/ml and 1.0×10^9^ cfu/ml were considered as *ad libitum* (AL) and dietary restriction (DR), respectively. Consistent with published results using other DR protocols such as *eat-2* [52], liquid DR [53],[54] and food deprivation [55], lifespan extension by msDR does not require DAF-16 (Figure S1). Previous studies indicated that DAF-16 is required for lifespan extension by sDR [49] and is partially required for that by intermittent fasting (IF), another method of DR [56]. The reason for the different involvement of DAF-16 between sDR and msDR is unknown, but it could be due to the timing of DR induction or the differences in bacterial growth between the two methods.

**Figure 3:**
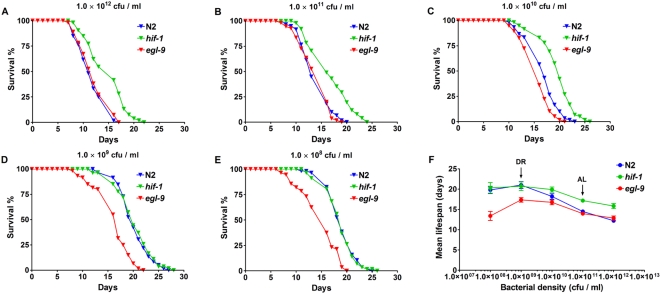
HIF-1 modulates lifespan extension by DR. (A–E) Survival curves and (F) mean lifespan of N2, *hif-1* and *egl-9* animals fed with *E. coli* at different concentrations (1.0×10^8^ to 1.0×10^12^ cfu/ml) during adulthood. The *hif-1* mutant shows lifespan extension under AL and higher bacterial concentrations but not under DR. *egl-9* animals show diminished lifespan extension under DR, but they show lifespan similar to that of N2 animals under AL conditions. Each of the lifespan experiments was performed three times with consistent results. Quantitative data and statistical analyses for the experiments shown here and the repeated experiments are included in Table 2 and Table S2, respectively.

We then examined longevity phenotypes of *hif-1* and *egl-9* mutants under different nutrient conditions. The *hif-1* mutation extended lifespan under AL but did not cause further lifespan extension under DR, whereas lifespan extension under DR was diminished by a mutation in *egl-9* (Figure 3; Table 2 and S2). We used both a random effects linear model [57] and a generalized estimating equation approach [58] to test whether the curve slopes for each mutant (*hif-1* and *egl-9*) are identical to that of N2 in Figure 3F. These methods showed that both *hif-1* and *egl-9* mutants have significantly reduced slopes in mean lifespan versus food concentrations relative to N2 (*p*<0.05). Thus, our data suggest that under high nutrient status, decreased HIF-1 levels may cause a shift to the DR state, and overexpression of HIF-1 partially inhibits the lifespan extension effect of DR. This phenotype is unlikely to be caused by behavior defects of the *hif-1* mutant since *hif-1* animals have a normal brood size and pumping rate (Figure S2).

**Table 2:** HIF-1 and IRE-1 modulate lifespan extension by DR.

Genotype	Food conc. (cfu/ml)	Mean lifespan a	Percent of control b	n c	*p*-value vs. control d
N2	1.0×10^12^	12.2±0.4	−	60	−
N2	1.0×10^11^	14.4±0.5	−	60	−
N2	1.0×10^10^	18.4±1.0	−	60	−
N2	1.0×10^9^	21.2±1.3	−	59	−
N2	1.0×10^8^	19.4±1.5	−	57	−
*hif-1(ia04)*	1.0×10^12^	15.8±1.1	130%	53	<0.0001
*hif-1(ia04)*	1.0×10^11^	17.2±0.5	119%	51	<0.0001
*hif-1(ia04)*	1.0×10^10^	19.9±1.1	108%	59	<0.0001
*hif-1(ia04)*	1.0×10^9^	20.8±2.0	98%	54	0.0685
*hif-1(ia04)*	1.0×10^8^	20.3±2.3	105%	57	0.0942
*egl-9(sa307)*	1.0×10^12^	12.9±0.9	106%	55	0.4297
*egl-9(sa307)*	1.0×10^11^	14.0±0.6	97%	55	0.7956
*egl-9(sa307)*	1.0×10^10^	16.7±1.0	91%	58	0.0045
*egl-9(sa307)*	1.0×10^9^	17.4±0.9	82%	60	<0.0001
*egl-9(sa307)*	1.0×10^8^	13.4±1.9	69%	56	<0.0001
*ire-1(v33)*	1.0×10^12^	9.4±0.5	77%	60	<0.0001
*ire-1(v33)*	1.0×10^11^	9.8±0.4	68%	59	<0.0001
*ire-1(v33)*	1.0×10^10^	10.2±0.2	55%	60	<0.0001
*ire-1(v33)*	1.0×10^9^	11.4±0.7	54%	60	<0.0001
*ire-1(v33)*	1.0×10^8^	10.5±0.6	54%	55	<0.0001
N2	− e	14.6±0.9	−	94	−
*egl-9(sa307)*	− e	14.5±1.0	99%	68	0.5532
*eat-2(ad1116)*	− e	18.2±0.1	125%	78	<0.0001
*eat-2(ad1116);egl-9(sa307)* f	− e	15.0±0.8	103%	99	0.1746

The lifespan experiments were performed for multiple times with consistent results. Data for representative experiments are shown in this table, and data for repeated experiments are shown in Table S2.

a average lifespan±standard deviation in days.

b changes in mean lifespan compared to N2 growing at the same food concentration.

c numbers of animals scored.

d *p*-values were calculated for log-rank tests by comparison to N2 growing at the same food concentration.

e animals were treated with *E. coli* OP50 food under standard lab conditions.

f log-rank tests: *eat-2* vs. *eat-2; egl-9*, *p*<0.0001.

**Figure 4:**
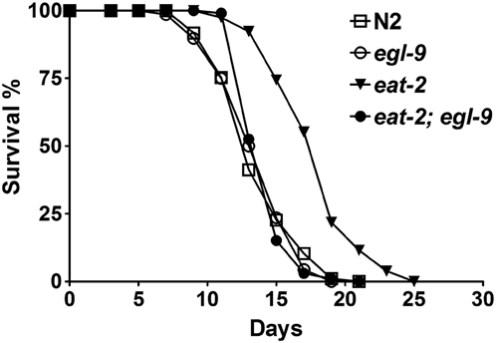
*egl-9* suppresses lifespan extension by the *eat-2* mutation. The lifespan experiments were performed twice with consistent results. Quantitative data and statistical analyses for the experiments shown here and the repeated experiments are included in Table 2 and Table S2, respectively.

Recent studies have shown that various protocols of DR extend lifespan through different mechanisms in *C. elegans* [49], [53]–[55],[59]. To further test whether HIF-1 is involved in DR-mediated lifespan extension, we examined the effect of *egl-9* on another form of DR using the *eat-2* mutant. *eat-2* encodes a subunit of nicotinic acetylcholine receptor, and mutations in *eat-2* cause significantly reduced pharyngeal pumping and extended lifespan. *eat-2* mutants have been widely used as a genetic mimic of DR in *C. elegans* [52]. We made the *eat-2; egl-9* double mutant and examined the adult lifespan. The *egl-9* mutation significantly suppressed the lifespan extension by a strong loss-of-function allele of *eat-2* (Figure 4; Table 2 and S2). Our results suggest that EGL-9 is an important regulator of longevity due to a genetic mimic of DR by the *eat-2* mutant.

It has been shown that the FOXA transcription factor PHA-4 is required for lifespan extension by liquid DR [54] and by a mutation in *rsks-1* [60]. To characterize the genetic interaction between *hif-1* and *pha-4*, we tested lifespan of N2 and *hif-1* animals treated with either control RNAi or *pha-4* RNAi. We found that *pha-4* RNAi slightly reduced lifespan in both N2 and *hif-1* backgrounds, but *hif-1* extended lifespan of animals treated with control or *pha-4* RNAi to a similar level (Figure S3). Thus, unlike *rsks-1*, lifespan extension by *hif-1* does not require PHA-4, suggesting HIF-1 is not the only downstream effector of RSKS-1.

### HIF-1 Functions in Specific Neurons and Muscles to Regulate DR-Dependent Lifespan Extension

A fundamental question about DR is how animals sense reduced nutrients in the environment and adjust physiology of the whole organism for extended survival. Previous studies suggest that the intercellular communication between the nutrient sensing and the major metabolic tissues coordinate physiological changes upon DR [53]. To define the sites where HIF-1 acts to regulate DR-dependent lifespan extension, we utilized previously published transgenic animals that express *egl-9* cDNA in various tissues in the *egl-9* mutant background [41].

**Figure 5:**
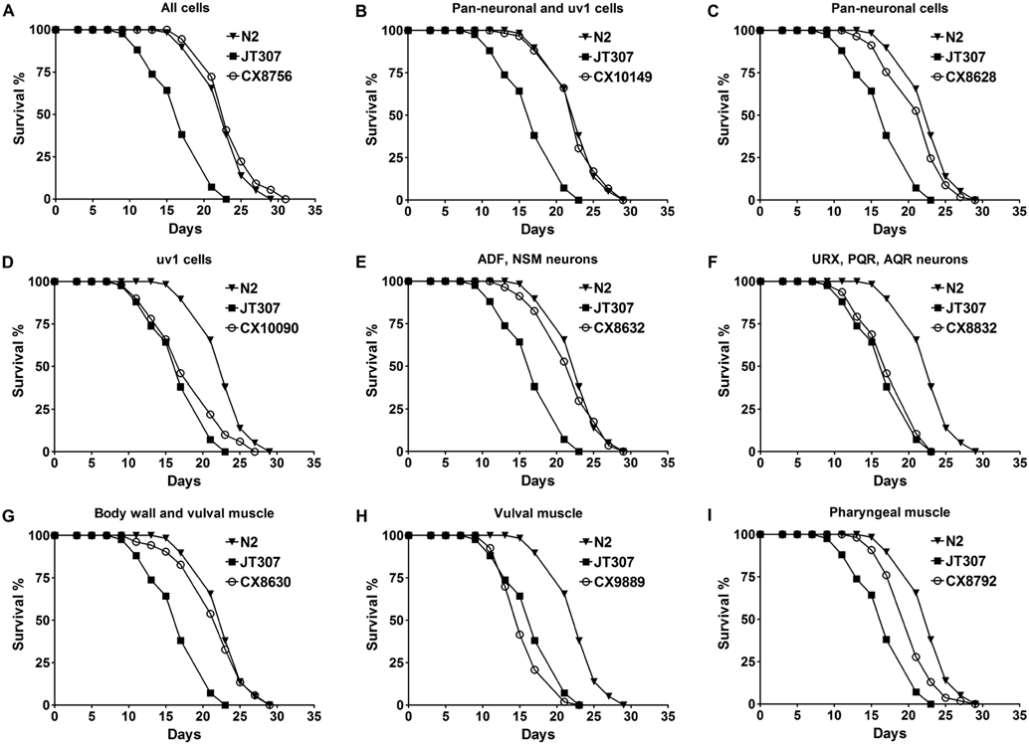
HIF-1 functions in specific neurons and muscles to regulate lifespan extension by DR. Lifespan of wild-type N2, JT307 *egl-9(sa307)* and *egl-9* animals with various tissue-specific promoters driving *egl-9* cDNA (CX strains) was measured under DR to determine the tissues where expression of *egl-9* rescues the lack of full lifespan extension by DR. Tissues where *egl-9* expression is restored were (A) all cells, (B) pan-neuronal and uv1 cells, (C) pan-neuronal cells, (D) uv1 cells, (E) ADF, NSM neurons, (F) URX, AQR, PQR neurons, (G) body wall and vulval muscles, (H) vulval muscle, and (I) pharyngeal muscle. Each of the lifespan experiments was performed twice with consistent results. Quantitative data and statistical analyses for the experiments shown here and the repeated experiments are included in Table 3 and Table S3, respectively.

Expression of the *egl-9* cDNA from its endogenous promoter, which drives *egl-9* expression in virtually all cells, completely rescued the shortened lifespan of *egl-9* under DR (Figure 5A, Table 3 and S3). Simultaneous expression of *egl-9* in pan-neuronal and uv1 uterine-vulval cells also rescued the mutant phenotype (Figure 5B, Table 3 and S3). Pan-neuronal expression alone was sufficient for the rescue (Figure 5C, Table 3 and S3), whereas uv1 cell expression showed very little rescuing effect (Figure 5D, Table 3). Further analyses indicated that *egl-9* expression in the serotonergic subset (ADF, NSM) rather than the soluble guanylate cyclase (sGC) subset (URX, AQR, PQR) of neurons that regulate the oxygen preference phenotype [61] was required for the lifespan extension by DR (Figure 5E and 5F; Table 3 and S3).

**Table 3:** HIF-1 functions in specific neurons and muscles to modulate DR-dependent lifespan extension.

Strain	Genotype	Mean lifespan a	Percent of control b	n c	*p*-value vs. N2 d	*p*-value vs. JT307 e
N2		21.8±1.7	−	58	−	<0.0001
JT307	*egl-9(sa307)*	16.9±0.3	78%	42	<0.0001	−
CX8756	*egl-9 (sa307); kyEx1593 [egl-9::egl-9::gfp]*	22.6±1.7	104%	54	0.1905	<0.0001
CX10149	*egl-9 (sa307); kyEx2321 [H20::egl-9::gfp, tdc-1::egl-9::gfp]*	21.5±1.8	99%	59	0.9522	<0.0001
CX8628	*egl-9 (sa307); kyEx1525 [H20::egl-9::gfp]*	20.5±1.5	94%	57	0.0590	<0.0001
CX10090	*egl-9 (sa307); kyEx2288 [tdc-1::egl-9::gfp]*	18.0±0.3	83%	50	<0.0001	0.1017
CX8632	*egl-9 (sa307); kyEx1529 [tph-1::egl-9::gfp]*	21.4±1.1	98%	57	0.4045	<0.0001
CX8832	*egl-9 (sa307); kyEx1639 [gcy-36::egl-9::gfp]*	17.6±0.3	81%	48	<0.0001	0.4079
CX8630	*egl-9 (sa307); kyEx1527 [myo-3::egl-9::gfp]*	21.3±1.1	98%	52	0.4647	<0.0001
CX9889	*egl-9 (sa307); kyEx2215 [hum-5::egl-9::gfp]*	14.8±1.6	68%	53	<0.0001	0.0489
CX8792	*egl-9 (sa307); kyEx1616 [myo-2::egl-9::gfp]*	20.0±1.1	92%	54	0.0002	<0.0001

Lifespan of N2, JT307 *egl-9(sa307)* and *egl-9* animals with various tissue-specific promoters driving *egl-9* cDNA (CX strains) was measured under DR. Experiments were performed twice with consistent results. Data for representative experiments are shown in this table, and data for repeated experiments are shown in Table S3.

a average lifespan±standard deviation in days.

b changes in mean lifespan compared to N2.

c numbers of animals scored.

d *p*-values were calculated for log-rank tests by comparison to N2.

e *p*-values were calculated for log-rank tests by comparison to JT307 *egl-9(sa307)*.

The *egl-9* cDNA expression driven by the *myo-3* promoter in body wall and vulval muscles also rescued the mutant phenotype (Figure 5G; Table 3 and S3). Vulval muscle expression alone did not rescue (Figure 5H; Table 3 and S3), suggesting that *egl-9* expression in body wall muscle, in addition to serotonergic neurons, is important for HIF-1-mediated lifespan extension via DR. The muscle tissue plays an important role in *C. elegans* aging. Previous studies have shown an age-related decline in the muscle structure and function in *C. elegans*, resembling human sarcopenia [62]. Pharyngeal muscle expression of *egl-9* by the *myo-2* promoter partially rescued the mutant (Figure 5I; Table 3 and S3). The pharynx is the food intake organ, and *myo-2* is a direct target of the PHA-4/FOXA transcription factor [63], which has been shown to be important for DR-dependent lifespan extension in *C. elegans* [54]. Expression of *egl-9* in other tissues, including the hypodermis, XXX endocrine cells, pharyngeal gland and pharyngeal marginal cells, did not rescue the lifespan phenotype under DR (data not shown). We also tested lifespan phenotypes of these transgenic animals under AL conditions. Tissue-specific rescue of *egl-9* did not significantly affect lifespan under AL (Figure S4; Table S4).

A recent study showed that HIF-1 acts both in neurons (ADF, NSM, URX, AQR, and PQR) and in gonadal endocrine cells (uv1) to regulate oxygen preference in *C. elegans* [41]. Our results indicate that HIF-1 acts in multiple cell types to modulate the longevity phenotypes of DR, and the lifespan and oxygen sensing effects of HIF-1 are determined by partially overlapped cell types.

### HIF-1 Functions through the IRE-1 ER Stress Pathway to Modulate Lifespan Extension by DR

ER stress is caused by a mismatch between the load of unfolded/misfolded proteins to ER and the capacity of the cellular machinery to cope with this load [64]. ER stress activates the unfolded protein response (UPR) through three signaling pathways transduced by IRE1, PERK, and ATF6. We speculated that DR and the *hif-1* mutant might extend lifespan through ER stress pathways since high nutrients have been shown to increase ER stress in rodents and activation of TOR also leads to increased ER stress [65]. Furthermore, the lifespan extension by mutations in the yeast TOR pathway is mediated through GCN4, which regulates the UPR during ER stress [17],[18].

**Figure 6:**
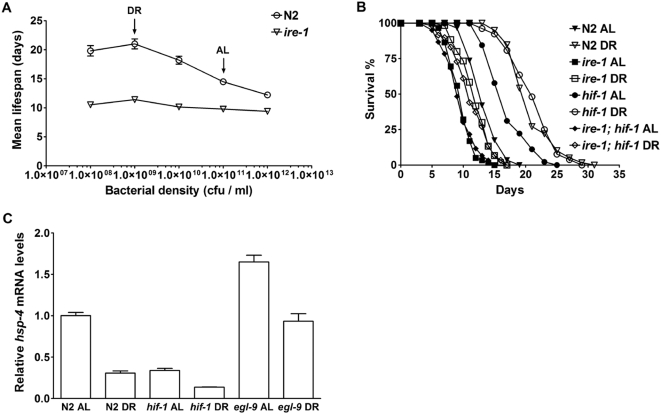
HIF-1 functions through the IRE-1 ER stress pathway to modulate lifespan. (A) IRE-1 modulates lifespan extension by DR. *ire-1(v33)* mutant animals show significantly reduced changes in lifespan upon nutrient manipulation. (B) *ire-1(v33)* fully suppresses lifespan extension by *hif-1* under both AL and DR conditions. Each of the lifespan experiments was performed multiple times with consistent results. Quantitative data and statistical analyses for the experiments shown here are included in Table 2 and Table 4. Analyses for the repeated experiments are included in Table S2 and Table S5. (C) *hsp-4* mRNA levels are regulated by DR and HIF-1. DR reduces *hsp-4* transcription in N2, *hif-1* and *egl-9* (*p*<0.05, t-test). Overexpression of HIF-1 due to the *egl-9(sa307)* mutation results in increased *hsp-4* transcription under both AL and DR conditions (*p*<0.01, t-test). qRT-PCR experiments were performed twice with consistent results using two independent RNA preparations.

We first examined whether the ER stress pathway is also involved in the lifespan extension by DR in *C. elegans*. *ire-1* encodes an ER transmembrane protein that senses misfolded proteins in the ER lumen and then activates the downstream transcription factor XBP-1 to regulate target gene expression and reduce ER stress [64]. We measured lifespan of a deletion mutant of *ire-1* with different concentrations of bacterial food. The *ire-1* mutant showed significantly reduced lifespan extension by DR (Figure 6A; Table 2 and S2). Using both a random effects linear model [57] and a generalized estimating equation approach [58], we found that the *ire-1* mutant has significantly reduced declines in mean lifespan versus food concentrations relative to N2 (*p*<0.05). Next, we tested whether the lifespan extension by *hif-1* is impacted by the ER stress pathway by measuring lifespan of the *ire-1; hif-1* double mutant. The *ire-1* mutation fully suppressed lifespan extension by *hif-1* both under AL and under DR conditions (Figure 6B; Table 4 and S5). Although the *ire-1* mutant is short-lived, the suppression of *hif-1* lifespan by *ire-1* is not due to sickness in general, but is specific for this longevity pathway. Previous studies showed that inhibition of the translation initiation factor-4G (*ifg-1*) extends lifespan in *C. elegans* [9],[12],[13]. *ifg-1* RNAi and the *hif-1* mutation have additive effects on lifespan (data not shown), suggesting they modulate longevity through different mechanisms. We observed that lifespan extension by *ifg-1* RNAi is not dependent on *ire-1* (Figure S5). We also found that RNAi knocking-down of *xbp-1*, an essential transcription factor downstream of IRE-1 for ER stress response, suppressed the lifespan extension by *hif-1* (Figure S6).

**Table 4:** IRE-1 is required for lifespan extension by *hif-1*.

Genotype	Food conc. (cfu/ml)	Mean lifespan a	Percent of control b	n c	*p*-value vs. control d
N2	1.0×10^11^	14.4±0.9	−	57	−
N2	1.0×10^9^	20.6±0.6	−	59	−
*ire-1(v33)*	1.0×10^11^	10.0±0.4	69%	59	<0.0001
*ire-1(v33)*	1.0×10^9^	11.8±0.1	57%	60	<0.0001
*hif-1(ia04)*	1.0×10^11^	17.5±0.4	122%	45	<0.0001
*hif-1(ia04)*	1.0×10^9^	20.3±1.5	99%	52	0.6311
*ire-1(v33); hif-1(ia04)* e	1.0×10^11^	9.2±0.5	64%	60	<0.0001
*ire-1(v33); hif-1(ia04)* f	1.0×10^9^	11.6±0.4	56%	59	<0.0001

The lifespan experiments were performed for twice with consistent results. Data for representative experiments are shown in this table, and data for repeated experiments are shown in Table S5.

a average lifespan±standard deviation in days.

b changes in mean lifespan compared to N2 growing at the same food concentration.

c numbers of animals scored.

d *p*-values were calculated for log-rank tests by comparison to N2 growing at the same food concentration.

e log-rank test: *ire-1* AL vs. *ire-1; hif-1* AL, *p* = 0.9076.

f log-rank test: *ire-1* DR vs. *ire-1; hif-1* DR, *p* = 0.5445.

*pek-1* encodes the *C. elegans* PERK homolog that functions in another branch of ER stress signaling. A deletion mutant of *pek-1* has a normal lifespan, and it does not affect *hif-1* lifespan both under AL and under DR conditions (Figure S7). Taken together, our results indicate that the IRE-1 ER stress pathway is a key effector of both *hif-1* and DR with respect to lifespan extension.

In order to determine whether HIF-1 modulates ER stress in response to nutritional variations, we examined the mRNA levels of *hsp-4*, which encodes an ER chaperone BiP ortholog in *C. elegans*. Under ER stress, *hsp-4* transcription increases dramatically, and it is widely used as an indicator of unfolded/misfolded protein overload [66]. We used quantitative RT-PCR (qRT-PCR) to measure *hsp-4* mRNA levels in wild-type N2, *hif-1*, and *egl-9* animals under different nutrient conditions. DR significantly reduced *hsp-4* transcription (Figure 6C), supporting the hypothesis that high nutrient levels correlate with high ER stress. The *hif-1* mutant under AL, which has extended lifespan, also showed reduced *hsp-4* mRNA compared to N2 under the same condition. With the DR treatment, *egl-9* animals, which have a shortened lifespan, showed higher *hsp-4* mRNA levels than N2 and *hif-1* (Figure 6C). C14B9.2 encodes a protein disulfide isomerase, and it was identified as a target of the IRE-1 pathway in *C. elegans* from previous studies [67]. Under ER stress, C14B9.2 mRNA levels increase dramatically, and the increased transcription is dependent on IRE-1 and XBP-1, but not PEK-1 [67]. Using qRT-PCR, we found that DR reduces C14B9.2 transcription, and *egl-9* animals have increased C14B9.2 mRNA levels under DR (Figure S8). Thus, lower mRNA levels of ER stress reporters, which indicate reduced ER-associated protein misfolding, accompanied genetic and environmental conditions that extend lifespan. These results support the role for ER signaling in lifespan extension both by DR and by *hif-1*. Since the ER stress markers do not totally correlate with lifespan phenotypes, e.g., *egl-9* under AL has higher *hsp-4* and C14B9.2 mRNA levels but a normal lifespan, there might be ER stress-independent factors functioning downstream of HIF-1 in lifespan determination.

**Figure 7:**
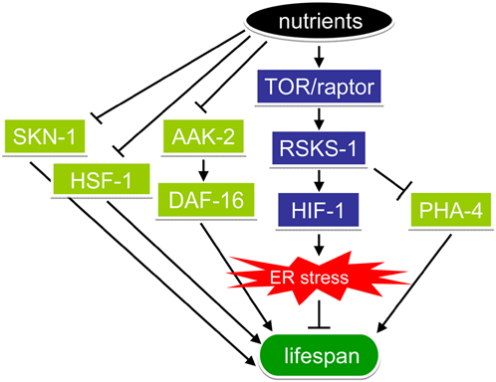
A genetic model depicting the modulation of lifespan by nutrients, HIF-1 and ER stress in *C. elegans*. High nutrients activate HIF-1 through the TOR-S6K pathway, which leads to increased ER stress and shortened lifespan. Other regulators such as PHA-4, SKN-1, AAK-2, DAF-16 and HSF-1 may function in parallel to HIF-1 to modulate DR-induced longevity phenotypes.

We propose a genetic model that depicts a pathway of lifespan modulation by nutrients which links the TOR pathway, HIF-1 and ER stress in *C. elegans* (Figure 7). This model indicates that HIF-1 acts downstream of the nutrient-responsive TOR-S6K pathway, as *egl-9* animals with elevated HIF-1 show diminished lifespan extension both under DR and by a mutation in S6K. Nutrients modulate ER stress, and the importance of ER signaling in determining lifespan is demonstrated by the finding that *ire-1* not only suppresses HIF-1-dependent lifespan extension, but also shows significantly diminished DR-dependent lifespan extension. We also find that reducing nutrient intake lessens unfolded protein damage in ER as measured by ER stress markers in *C. elegans*. Interestingly, inhibition of HIF-1, which extends lifespan under the AL condition, was also able to reduce the ER stress under this condition. Conversely, the *egl-9* mutants, which fail to show maximal lifespan extension by DR, had elevated levels of the ER stress markers under DR. Together, these experiments suggest that HIF-1 functions through the IRE-1 ER stress pathway to modulate lifespan extension by DR. Since DR-dependent lifespan extension cannot be fully suppressed by either increased HIF-1 or the mutation in *ire-1*, there are potentially other genes that function in parallel to *hif-1* to determine DR-dependent lifespan extension.

## Discussion

Previous studies have identified HIF-1 as a key regulator in various cellular processes, including stress resistance, glucose metabolism, angiogenesis and cell death. Despite the well-characterized HIF-1 functions in age-associated physiological processes and the regulation of HIF-1 by known lifespan determinants, such as TOR and S6K, it has not been clear whether HIF-1 is involved in organismal aging. In this study, we found that a deletion mutant of *hif-1* shows significant lifespan extension in *C. elegans*. Genetic epistasis experiments indicate that HIF-1 functions downstream of the nutrient-responsive TOR-S6K pathway to modulate lifespan. Using a modified sDR regime, we found that HIF-1 is involved in DR-mediated lifespan regulation, with overexpression of HIF-1 diminishes lifespan extension under DR.

Recent studies in *C. elegans* have identified novel genetic pathways that determine the beneficial effects of DR [49], [53]–[55],[68]. Using different regimens to restrict nutrients intake, previous studies have identified key regulators of DR-dependent lifespan extension, including transcription factors PHA-4, SKN-1, HSF-1 and the cellular energy homeostasis regulator AMP-activated protein kinase (AMPK). It is very likely that the maximal lifespan extension by DR is not achieved by regulating a single genetic pathway but that multiple pathways act together to mediate the lifespan effect of DR [69],[70]. The TOR pathway has been shown to play important roles in DR-dependent lifespan extension in *D. melanogaster* [6] and *S. cerevisiae* [7]. Our study underscores the importance of the TOR-S6K pathway as a conserved mediator of lifespan extension by DR in multiple species.

One of the most important questions about DR is how animals sense reduced nutrients to adjust gene expression, metabolism and behavior for extended survival. It has been shown that the SKN-1 transcription factor functions in ASI neurons to mediate liquid DR-induced longevity by an endocrine mechanism [53]. Using transgenic animals that express *egl-9* cDNA with various tissue-specific promoters in the *egl-9* mutant background [41], we found that restoring EGL-9 function in serotonergic neurons (ADF, NSM), body wall muscle and pharyngeal muscle can rescue lifespan phenotypes of *egl-9* under DR. Serotonin is a neurotransmitter that regulates feeding, reproduction, and fat metabolism in *C. elegans*. [71],[72]. Recently, serotonin signaling has also been connected to aging in *C. elegans* [73],[74]. It will be interesting to examine whether HIF-1 regulates serotonin signaling, and whether serotonin signaling is involved in DR-dependent lifespan extension in *C. elegans*. A previous study has shown that muscle decline is one of the major physiological causes of *C. elegans* aging [62]. Whether DR and HIF-1 affect muscle structure and function during aging needs to be further investigated. The pharynx is the food intake organ of *C. elegans*. The FOXA transcription factor PHA-4 plays essential roles in pharyngeal development [75]. Interestingly, PHA-4 is required for lifespan extension by DR [54] and by a mutation in S6K [76], but lifespan extension by *hif-1* is not dependent on PHA-4. These experiments suggest that there may be multiple pathways downstream of TOR/S6K that mediate lifespan extension in response to DR.

Abundant evidence has indicated that ER stress and protein homeostasis are important for aging [17],[59],[77],[78]. ER stress is caused by an overload of unfolded/misfolded proteins to ER [64]. ER stress activates the unfolded protein response through three signaling pathways transduced by IRE1, PERK, and ATF6. We showed that lifespan extension by DR or *hif-1* requires functional IRE-1 and XBP-1 ER stress signaling. Reduced nutrients are associated with lower ER stress, which is consistent with findings in mice that increased nutrient intake or TOR activation is linked to increased ER stress [65],[79]. Our results implicating the role of ER stress in DR-mediated lifespan extension in *C. elegans* are also consistent with findings from *S. cerevisae*, in which the lifespan extension by DR was found to be partially dependent on GCN4 [17], which functions downstream of PERK in the unfolded protein response [80]. Our work describes a novel role for ER signaling in aging and DR-dependent lifespan extension, examination of which may help explain how protein homeostasis determines lifespan and age-related diseases.

DR has not only been shown to extend lifespan in rodents but also is one of the most robust methods to reduce tumorigenesis in mice [81]. However, the mechanisms by which DR causes this protection have not been elucidated. Our results implicate HIF-1 as a potential target in mediating the protective effects of DR on tumorigenesis in mammals. HIF-1 overexpression is frequently detected in various tumors due to intratumoral hypoxia and genetic mutations. HIF-1 owes its oncogenic properties to pleiotropic effects on a variety of cellular processes, including survival under hypoxia, angiogenesis, metastasis and glucose metabolism. Inhibition of HIF-1 has been proven to be an efficient way to prevent tumor growth, and HIF-1 is being extensively studied as an important target in cancer therapy. Our study suggests an important role for HIF-1 as an oncogene in the context of aging and nutrient sensing, which are both key risk factors in tumor formation.

## Materials and Methods

### Nematode Growth and Strains

Strains were cultured under standard lab condition as described [82]. Strains used in this work include N2, ZG31 *hif-1(ia04) V*, JT307 *egl-9(sa307) V*, GR1329 *daf-16(mgDf47) I*, CB1370 *daf-2(e1370) III*, DR1439 *unc-24(e138) daf-15(m634) IV/nT1[let-?(m435)] (IV;V)*, XA8223 *rsks-1(ok1255) III*, XA8206 *rsks-1(ok1255) III; hif-1(ia04) V*, XA8208 *rsks-1(ok1255) III; egl-9(sa307) V*, DA1116 *eat-2(ad1116) II*, PKL21 *eat-2(ad1116) II; egl-9(sa307) V*, CX8628 *egl-9 (sa307) V; kyEx1525 [H20::egl-9::gfp]*, CX8630 *egl-9 (sa307) V; kyEx1527 [myo-3::egl-9::gfp]*, CX8632 *egl-9 (sa307) V; kyEx1529 [tph-1::egl-9::gfp]*, CX8756 *egl-9 (sa307) V; kyEx1593 [egl-9::egl-9::gfp]*, CX8792 *egl-9 (sa307) V; kyEx1616 [myo-2::egl-9::gfp]*, CX8832, *egl-9 (sa307) V; kyEx1639 [gcy-36::egl-9::gfp]*, CX9646 *egl-9 (sa307) V; kyEx2109 [eak-4::egl-9::gfp]*, CX9778 *egl-9 (sa307) V; kyEx2159 [col-19::egl-9::gfp]*, CX9779 *egl-9 (sa307) V; kyEx2160 [hlh-6::egl-9::gfp]*, CX9807 *egl-9 (sa307) V; kyEx2161[ttx-1::egl-9::gfp]*, CX9889 *egl-9 (sa307) V; kyEx2215 [hum-5::egl-9::gfp]*, CX10002 *egl-9 (sa307) V; kyEx2254 [unc-31::egl-9::gfp]*, CX10090 *egl-9 (sa307) V; kyEx2288 [tdc-1::egl-9::gfp]*, CX10149 *egl-9 (sa307) V; kyEx2321 [H20::egl-9::gfp, tdc-1::egl-9::gfp]*, RE666 *ire-1(v33) II*, XA8234 *ire-1(v33) II; hif-1(ia04) V*, PKL3 *pek-1(ok275) X*, and PKL6 *hif-1(ia04) V; pek-1(ok275) X*.

### Dietary Restriction

The msDR method was modified from previously described [49]. Overnight culture of *E. coli* OP50 grown at 37°C was centrifuged at 3,000 rpm for 30 minutes to collect bacteria cells. The bacterial pellet was washed with the S buffer, and the bacterial concentration was adjusted to 1.0×10^12^ cfu/ml. Serial dilutions were performed to achieve bacterial concentrations of 1.0×10^11^, 1.0×10^10^, 1.0×10^9^, and 1.0×10^8^ cfu/ml. Diluted bacterial cultures were spotted onto DR agar plates, which were modified from the standard nematode growth media (NGM) plates by excluding peptone and increasing agar from 1.7% to 2.0%. Carbenicillin (50 µg/ml) was added to the agar plates to further prevent bacteria growth. Synchronized L4 larvae growing under standard lab conditions (NGM plates with OP50 food, 20°C) were transferred to fresh NGM plates with OP50 food and 5 µg/ml of FUdR, and were incubated at 25°C overnight. Day 1 adult animals were then transferred to DR agar plates seeded with OP50 at different concentrations.

In the first week of lifespan experiments and heat stress assays, 5-fluorodeoxyuridine (FUdR) at 50 µg/ml was also added into the agar plates to prevent progeny from hatching.

### Lifespan Assays

Lifespan assays using standard lab conditions were performed as previously described [16]. Late L4 larvae growing at 20°C were transferred to fresh NGM plates with FUdR (5 µg/ml) and incubated at 25°C. The first day of adulthood is Day 1 in survival curves. Animals were scored as alive, dead or lost every other day. Animals that failed to display touch-provoked movement were scored as dead. Animals that died from causes other than aging, such as sticking to the plate walls, internal hatching or bursting in the vulval region, were scored as lost. Animals were transferred to fresh plates every 3–6 days. All lifespan experiments were performed at 25°C. Survival curves were plotted and statistical analyses (log-rank tests) were performed using the Prism 4 software (Graphpad Software, Inc., San Diego, CA, USA).

### Brood Size Assays

Wild-type N2 L4 larvae growing at 20°C were transferred to plates seeded with *E. coli* food at different concentrations. Those animals were transferred every day to fresh plates and progeny produced during that 24-hour period were counted.

### Heat Stress Assays

Adult wild-type N2 animals grown on DR plates with bacterial food diluted at different concentrations for 48 hours since Day 1 adulthood were used for heat stress assays. The temperature was shifted from 20°C to 35°C and survival was scored. Animals that failed to display touch-provoked movement were scored as dead. Survival curves were plotted using the Prism 4 software (Graphpad Software, Inc., San Diego, CA, USA).

### Quantitative RT-PCR Assays

Adult N2, *hif-1*, and *egl-9* animals grown under either AL or DR for 5 days since Day 1 adulthood were collected for total RNA preparations using the Trizol reagent (Invitrogen). The first strand cDNA was synthesized using the reverse transcription system (Qiagen). SYBR Green dye (Quanta) was used for qRT-PCR. Reactions were performed in triplicate on an ABI Prism 7000 real-time PCR machine (Applied Biosystems). Relative-fold changes were calculated using the 2^−ΔΔCt^ method [83]. qRT-PCR experiments were performed twice with consistent results using two independent RNA preparations. The sequences of primers used were *act-1* forward, CAA TCC AAG AGA GGT ATC CTT ACC CTC; *act-1* reverse, GAG GAG GAC TGG GTG CTC TTC; *hsp-4* forward, GGA AGC ATA TGC CTA TCA GAT G; *hsp-4* reverse, CAG ATT CAA GTT CCT TCT TTT GC; C14B9.2 forward, GTT GTT CTC GCC AAG ATG GAC; and C14B9.2 reverse, GAT TGG TTC ACT CTT CTT TCC AGC.

## Supporting Information

Figure S1 DR-mediated lifespan extension is not dependent on DAF-16. Survival curves (A) and mean lifespan (B) of *daf-16(mgDf47)* animals fed with *E. coli* at different concentrations (1.0×10^8^ to 1.0×10^12^ cfu/ml) during adulthood. Mean lifespan was 10.1 days for 1.0×10^12^ cfu/ml (n = 59), 10.9 days for 1.0×10^11^ cfu/ml (n = 60), 13.0 days for 1.0×10^10^ cfu/ml (n = 59), 17.4 days for 1.0×10^9^ cfu/ml (n = 57), and 16.1 days for 1.0×10^8^ cfu/ml (n = 58). Log-rank tests: animals treated with different concentrations of food vs. AL (1.0×10^11^ cfu/ml), *p* = 0.0049 for 1.0×10^12^ cfu/ml, *p*<0.0001 for 1.0×10^10^ cfu/ml, 1.0×10^9^ cfu/ml and 1.0×10^8^ cfu/ml.
(2.79 MB TIF)

Figure S2 *hif-1* does not affect brood size and pumping rate. (A) The *hif-1(ia04)* mutant has a normal brood size. Average brood sizes were 275±27 for N2 and 285±18 for *hif-1*. t - test: *p* = 0.3350. Ten animals were examined in each genetic background. (B) The *hif-1(ia04)* mutant has a normal pumping rate. Average pumping rates (number of pharyngeal pumps per 20 seconds) of Day 2 adult animals were 90.0±10.1 for N2 and 90.7±12.2 for *hif-1*. t - test: *p* = 0.8904. Ten animals were examined for each genetic background.
(1.02 MB TIF)

Figure S3 Lifespan extension by *hif-1* is not dependent on *pha-4*. Mean life was 14.0 days for N2 with control RNAi (n = 96), 12.5 days for N2 with *pha-4* RNAi (n = 95), 17.8 days for *hif-1* with control RNAi (n = 103) and 16.5 days for *hif-1* with *pha-4* RNAi (n = 82). n, numbers of animals scored. *pha-4* RNAi reduces lifespan in both N2 and *hif-1* backgrounds (Log-rank tests: *p*<0.0001). However, *hif-1* extends lifespan to similar levels for both control RNAi and *pha-4* RNAi treated animals (*hif-1* with control RNAi vs. N2 with control RNAi: mean lifespan extension 27%, *p*<0.0001; *hif-1* with *pha-4* RNAi vs. N2 with *pha-4* RNAi: mean lifespan extension 32%, *p*<0.0001).
(0.89 MB TIF)

Figure S4 Tissue-specific rescue of *egl-9* does not significantly affect lifespan under AL. Lifespan of wild-type N2, JT307 *egl-9(sa307)* and *egl-9* animals with various tissue-specific promoters driving *egl-9* cDNA (CX strains) was measured under AL. Tissues where *egl-9* expression is restored were (A) all cells, (B) pan-neuronal and uv1 cells, (C) pan-neuronal cells, (D) uv1 cells, (E) ADF, NSM neurons, (F) URX, AQR, PQR neurons, (G) body wall and vulval muscles, (H) vulval muscle, and (I) pharyngeal muscle. Detailed statistical analyses are shown in Table S4.
(2.37 MB TIF)

Figure S5 *ire-1* does not suppress lifespan extension by *ifg-1* RNAi. Mean lifespan was 12.4 days for N2 with control RNAi (n = 115); 18.5 days for N2 with *ifg-1* RNAi (n = 91); 6.6 days for *ire-1* with control RNAi (n = 116); and 10.2 days for *ire-1* with *ifg-1* RNAi (n = 113). *ifg-1* RNAi extends N2 and *ire-1* lifespan by 49% and 55%, respectively. Log-rank test: *ire-1* with control RNAi vs. *ire-1* with *ifg-1* RNAi, *p*<0.0001. n, numbers of animals scored.
(0.93 MB TIF)

Figure S6 Lifespan extension by *hif-1* is suppressed by *xbp-1* RNAi. Mean life was 14.9 days for N2 with control RNAi (n = 134), 13.5 days for N2 with *xbp-1* RNAi (n = 135), 17.8 days for *hif-1* with control RNAi (n = 103) and 14.3 days for *hif-1* with *xbp-1* RNAi (n = 148). n, numbers of animals scored. Log-rank tests: N2 with control RNAi vs. N2 with *xbp-1* RNAi, *p*<0.0001; *hif-1* with control RNAi vs. *hif-1* with *xbp-1* RNAi, *p*<0.0001.
(0.89 MB TIF)

Figure S7 *pek-1* does not affect lifespan under both AL and DR conditions. Mean lifespan was 15.3 days for N2 AL (n = 57), 22.3 days for N2 DR (n = 55), 15.7 days for *pek-1* AL (n = 59), 22.1 days for *pek-1* DR (n = 59), 18.9 days for *hif-1* AL (n = 51), 22.2 days for *hif-1 DR* (n = 58), 17.8 days for *hif-1;pek-1* AL (n = 50), and 22.6 days for *hif-1;pek-1* DR (n = 56). n, numbers of animals scored. *pek-1* has no effects on lifespan in all genetic backgrounds and under different nutrient conditions (Log rank test: *p*>0.05).
(0.94 MB TIF)

Figure S8 C14B9.2 mRNA levels are regulated by DR and HIF-1. DR reduces C14B9.2 transcription in N2, *hif-1* and *egl-9* (*p*<0.05, t - test). Overexpression of HIF-1 due to the *egl-9(sa307)* mutation results in increased C14B9.2 transcription under both AL and DR conditions (*p*<0.01, t - test). qRT-PCR experiments were performed twice with consistent results using two independent RNA preparations.
(2.03 MB TIF)

Table S1 HIF-1 functions in the TOR-S6K pathway to modulate *C. elegans* lifespan.
(0.04 MB DOC)

Table S2 HIF-1 and IRE-1 mediate lifespan extension by DR.
(0.06 MB DOC)

Table S3 HIF-1 functions in specific neurons and muscles to modulate DR-dependent lifespan extension.
(0.04 MB DOC)

Table S4 Tissue-specific rescue of *egl-9* does not affect lifespan under AL.
(0.04 MB DOC)

Table S5 IRE-1 is required for lifespan extension by *hif-1*.
(0.03 MB DOC)
