# Supplementary figures and images for: HIF-1 Modulates Dietary Restriction-Mediated Lifespan Extension via IRE-1 in Caenorhabditis elegans
Source: PLoS Genet. 2009 May 22;5(5):e1000486. doi: 10.1371/journal.pgen.1000486 (PMC2676694; doi:10.1371/journal.pgen.1000486)

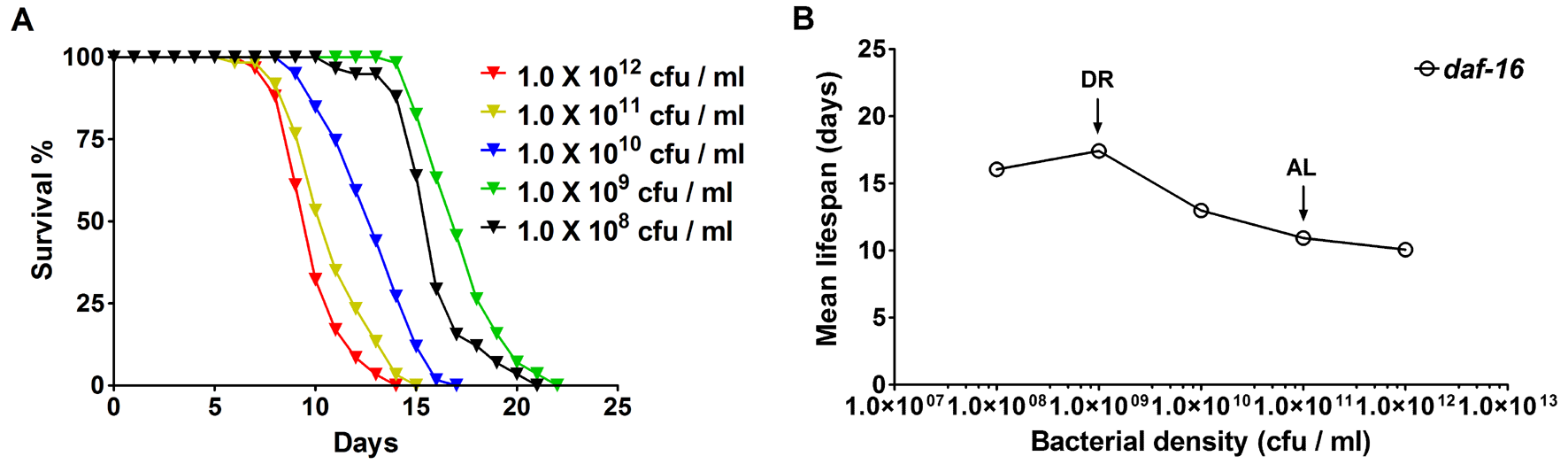

Supplement: Figure S1 — DR-mediated lifespan extension is not dependent on DAF-16. Survival curves (A) and mean lifespan (B) of daf-16(mgDf47) animals fed with E. coli at different concentrations (1.0×10^8^ to 1.0×10^12^ cfu/ml) during adulthood. Mean lifespan was 10.1 days for 1.0×10^12^ cfu/ml (n = 59), 10.9 days for 1.0×10^11^ cfu/ml (n = 60), 13.0 days for 1.0×10^10^ cfu/ml (n = 59), 17.4 days for 1.0×10^9^ cfu/ml (n = 57), and 16.1 days for 1.0×10^8^ cfu/ml (n = 58). Log-rank tests: animals treated with different concentrations of food vs. AL (1.0×10^11^ cfu/ml), p = 0.0049 for 1.0×10^12^ cfu/ml, p<0.0001 for 1.0×10^10^ cfu/ml, 1.0×10^9^ cfu/ml and 1.0×10^8^ cfu/ml. (2.79 MB TIF) [file pgen.1000486.s001.tif]

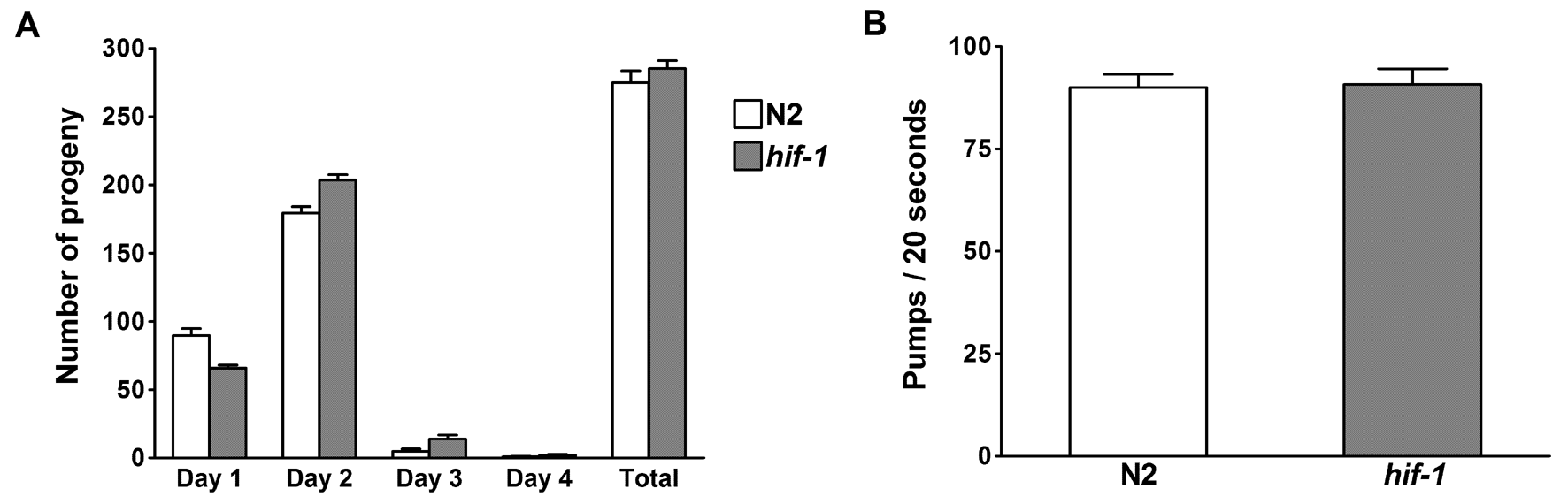

Supplement: Figure S2 — hif-1 does not affect brood size and pumping rate. (A) The hif-1(ia04) mutant has a normal brood size. Average brood sizes were 275±27 for N2 and 285±18 for hif-1. t - test: p = 0.3350. Ten animals were examined in each genetic background. (B) The hif-1(ia04) mutant has a normal pumping rate. Average pumping rates (number of pharyngeal pumps per 20 seconds) of Day 2 adult animals were 90.0±10.1 for N2 and 90.7±12.2 for hif-1. t - test: p = 0.8904. Ten animals were examined for each genetic background. (1.02 MB TIF) [file pgen.1000486.s002.tif]

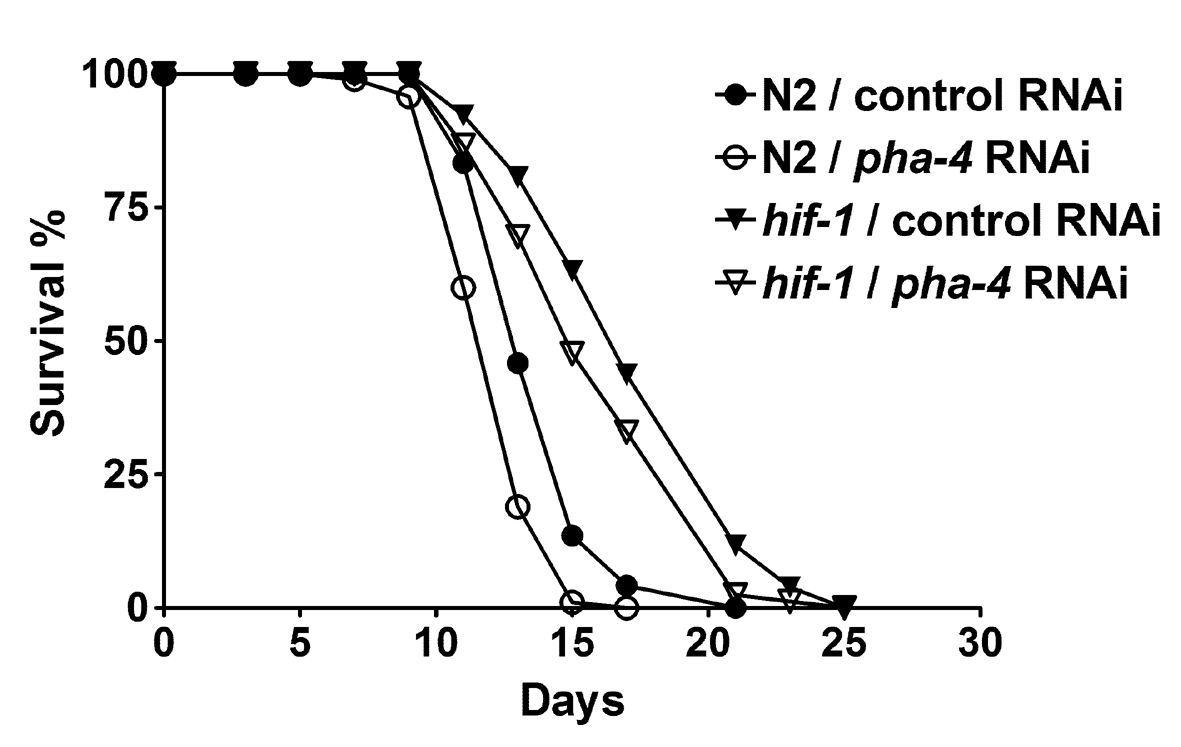

Supplement: Figure S3 — Lifespan extension by hif-1 is not dependent on pha-4. Mean life was 14.0 days for N2 with control RNAi (n = 96), 12.5 days for N2 with pha-4 RNAi (n = 95), 17.8 days for hif-1 with control RNAi (n = 103) and 16.5 days for hif-1 with pha-4 RNAi (n = 82). n, numbers of animals scored. pha-4 RNAi reduces lifespan in both N2 and hif-1 backgrounds (Log-rank tests: p<0.0001). However, hif-1 extends lifespan to similar levels for both control RNAi and pha-4 RNAi treated animals (hif-1 with control RNAi vs. N2 with control RNAi: mean lifespan extension 27%, p<0.0001; hif-1 with pha-4 RNAi vs. N2 with pha-4 RNAi: mean lifespan extension 32%, p<0.0001). (0.89 MB TIF) [file pgen.1000486.s003.tif]

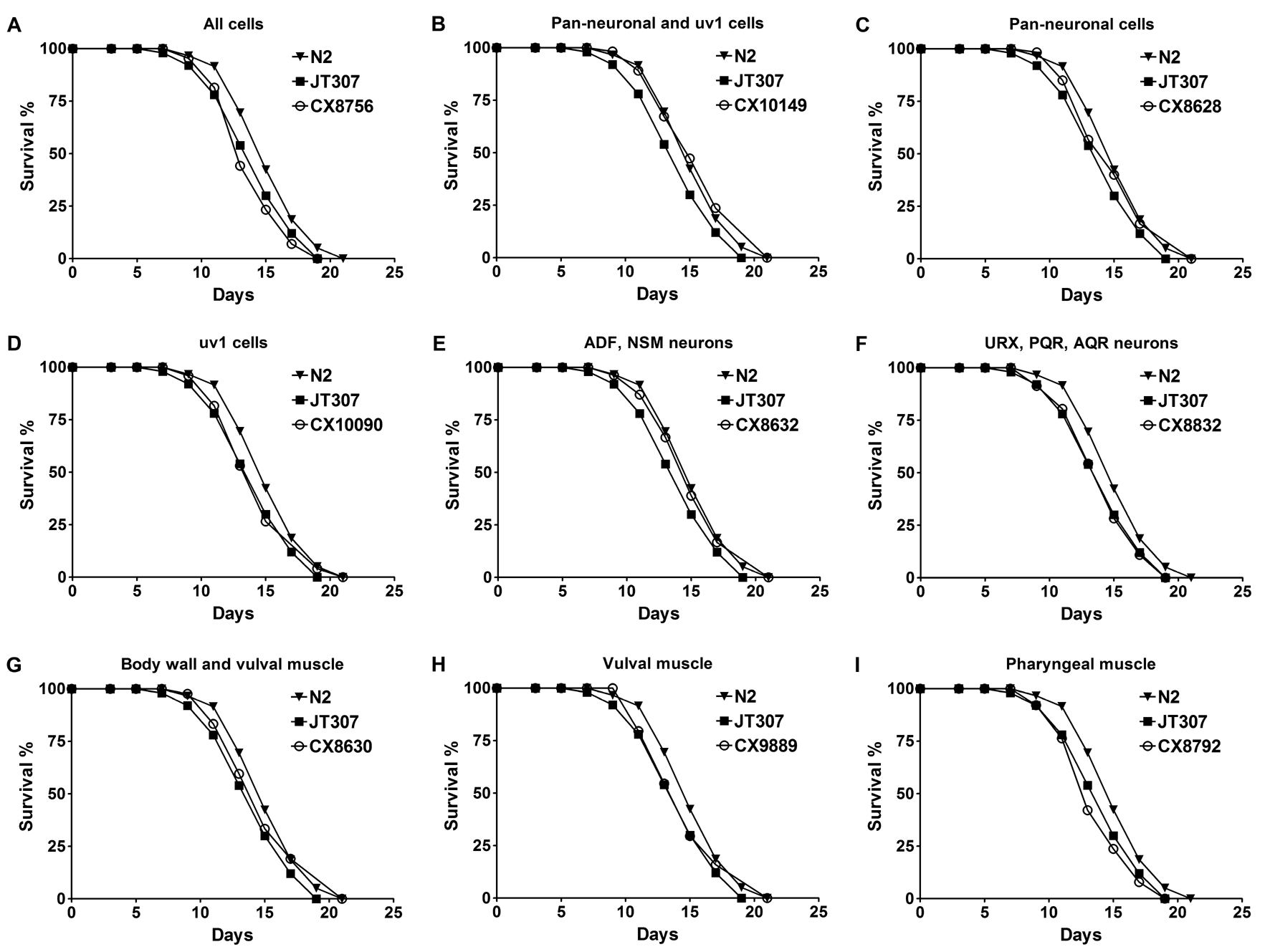

Supplement: Figure S4 — Tissue-specific rescue of egl-9 does not significantly affect lifespan under AL. Lifespan of wild-type N2, JT307 egl-9(sa307) and egl-9 animals with various tissue-specific promoters driving egl-9 cDNA (CX strains) was measured under AL. Tissues where egl-9 expression is restored were (A) all cells, (B) pan-neuronal and uv1 cells, (C) pan-neuronal cells, (D) uv1 cells, (E) ADF, NSM neurons, (F) URX, AQR, PQR neurons, (G) body wall and vulval muscles, (H) vulval muscle, and (I) pharyngeal muscle. Detailed statistical analyses are shown in Table S4. (2.37 MB TIF) [file pgen.1000486.s004.tif]

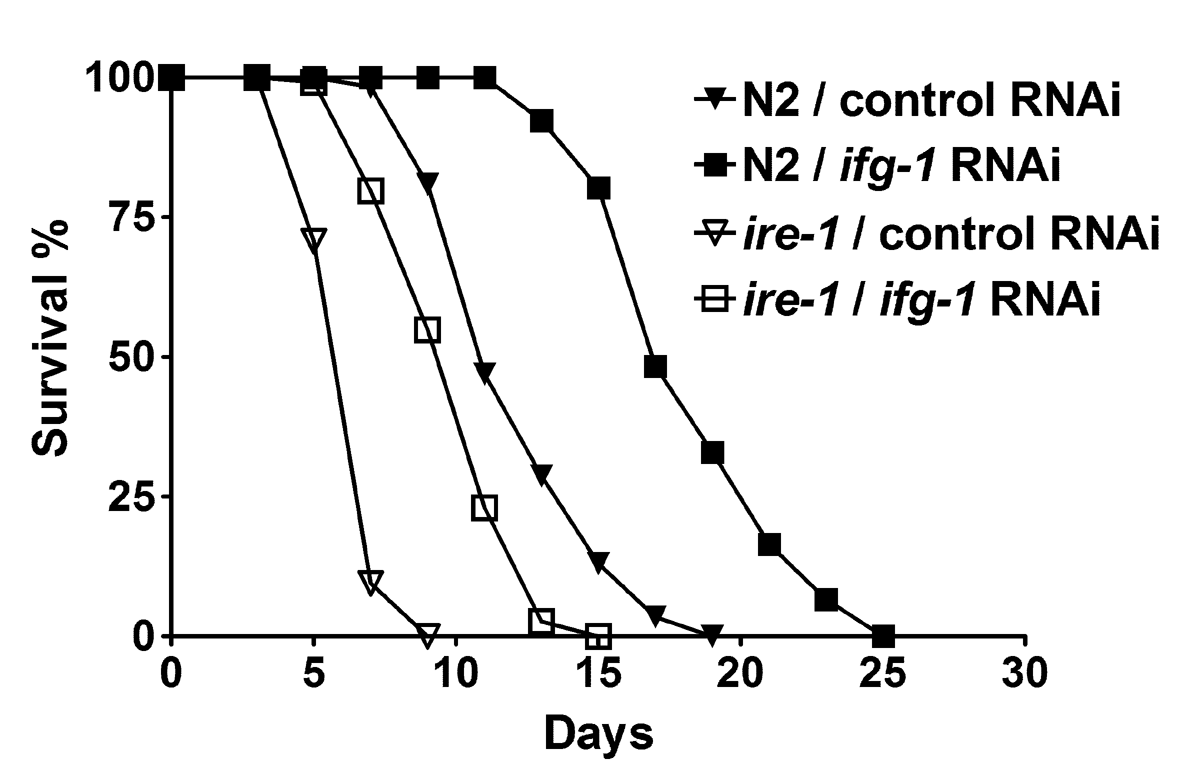

Supplement: Figure S5 — ire-1 does not suppress lifespan extension by ifg-1 RNAi. Mean lifespan was 12.4 days for N2 with control RNAi (n = 115); 18.5 days for N2 with ifg-1 RNAi (n = 91); 6.6 days for ire-1 with control RNAi (n = 116); and 10.2 days for ire-1 with ifg-1 RNAi (n = 113). ifg-1 RNAi extends N2 and ire-1 lifespan by 49% and 55%, respectively. Log-rank test: ire-1 with control RNAi vs. ire-1 with ifg-1 RNAi, p<0.0001. n, numbers of animals scored. (0.93 MB TIF) [file pgen.1000486.s005.tif]

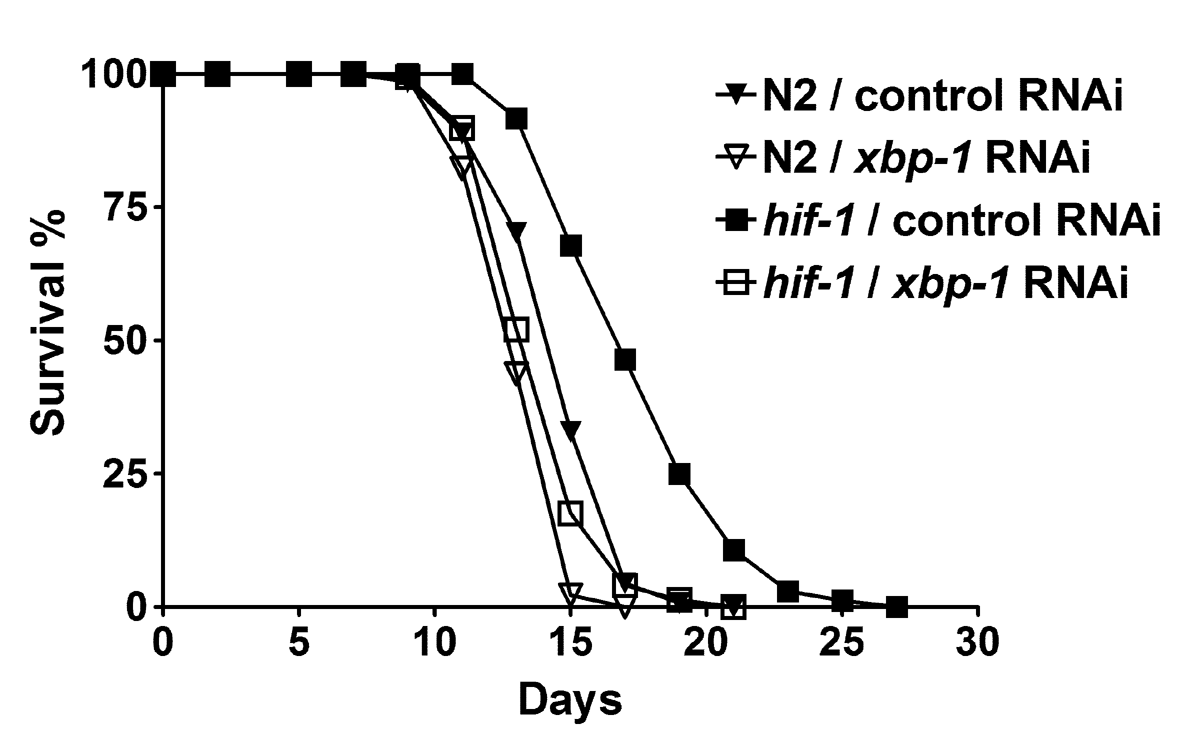

Supplement: Figure S6 — Lifespan extension by hif-1 is suppressed by xbp-1 RNAi. Mean life was 14.9 days for N2 with control RNAi (n = 134), 13.5 days for N2 with xbp-1 RNAi (n = 135), 17.8 days for hif-1 with control RNAi (n = 103) and 14.3 days for hif-1 with xbp-1 RNAi (n = 148). n, numbers of animals scored. Log-rank tests: N2 with control RNAi vs. N2 with xbp-1 RNAi, p<0.0001; hif-1 with control RNAi vs. hif-1 with xbp-1 RNAi, p<0.0001. (0.89 MB TIF) [file pgen.1000486.s006.tif]

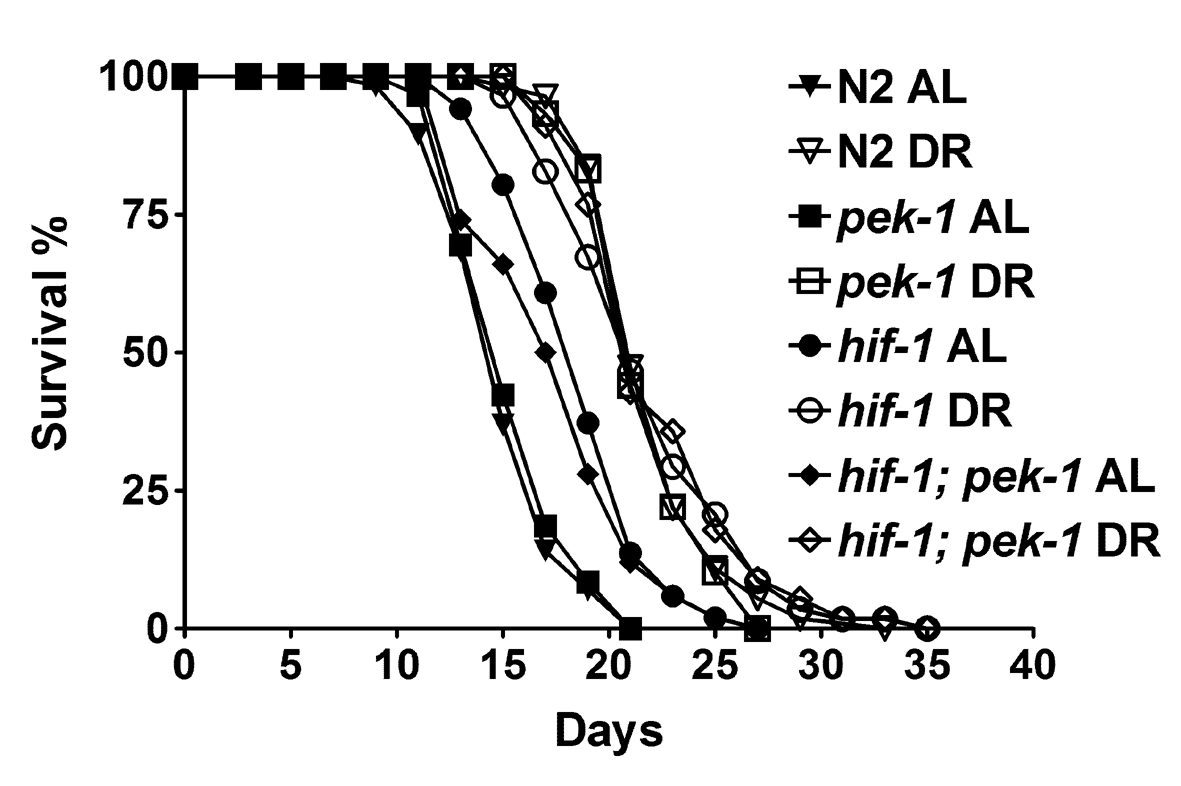

Supplement: Figure S7 — pek-1 does not affect lifespan under both AL and DR conditions. Mean lifespan was 15.3 days for N2 AL (n = 57), 22.3 days for N2 DR (n = 55), 15.7 days for pek-1 AL (n = 59), 22.1 days for pek-1 DR (n = 59), 18.9 days for hif-1 AL (n = 51), 22.2 days for hif-1 DR (n = 58), 17.8 days for hif-1;pek-1 AL (n = 50), and 22.6 days for hif-1;pek-1 DR (n = 56). n, numbers of animals scored. pek-1 has no effects on lifespan in all genetic backgrounds and under different nutrient conditions (Log rank test: p>0.05). (0.94 MB TIF) [file pgen.1000486.s007.tif]

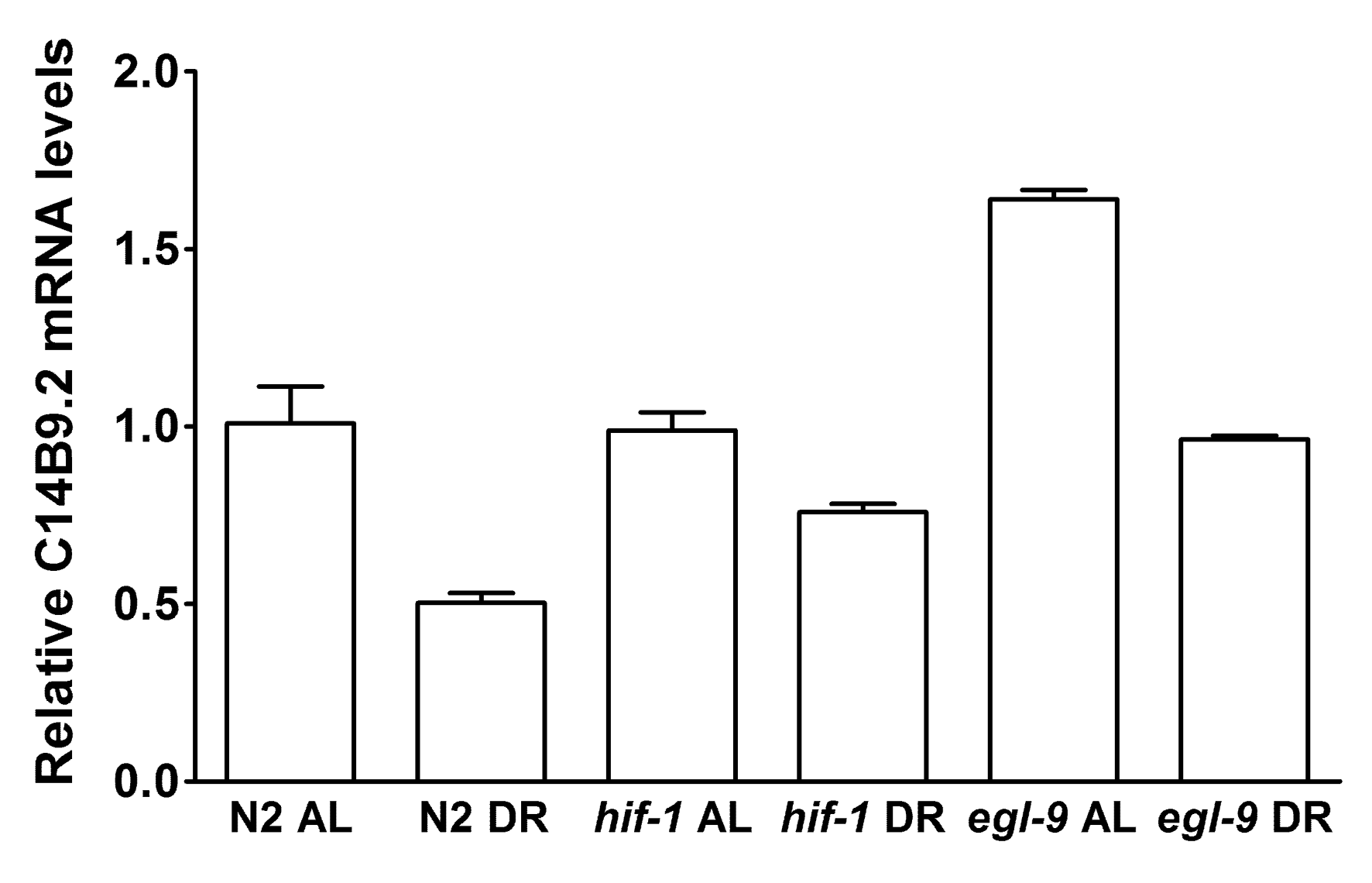

Supplement: Figure S8 — C14B9.2 mRNA levels are regulated by DR and HIF-1. DR reduces C14B9.2 transcription in N2, hif-1 and egl-9 (p<0.05, t - test). Overexpression of HIF-1 due to the egl-9(sa307) mutation results in increased C14B9.2 transcription under both AL and DR conditions (p<0.01, t - test). qRT-PCR experiments were performed twice with consistent results using two independent RNA preparations. (2.03 MB TIF) [file pgen.1000486.s008.tif]
