# Supplementary material for: HIF-1 Modulates Dietary Restriction-Mediated Lifespan Extension via IRE-1 in Caenorhabditis elegans
Source: PLoS Genet. 2009 May 22;5(5):e1000486. doi: 10.1371/journal.pgen.1000486 (PMC2676694; doi:10.1371/journal.pgen.1000486)
Supplement: Table S1 — HIF-1 functions in the TOR-S6K pathway to modulate C. elegans lifespan. (0.04 MB DOC) [file pgen.1000486.s009.doc]

**Table S1. HIF-1 functions in the TOR-S6K pathway to modulate *C. elegans* lifespan**

| **Genotype** | **RNAi** | **Mean lifespan ^a^** | **Percent of control ^b^** | **n ^c^** | ***p*-value vs. control ^d^** |
| --- | --- | --- | --- | --- | --- |
| N2 | − ^e^ | 14.4 | − | 117 | − |
| *hif-1(ia04)* | − ^e^ | 16.3 | 113% | 139 | <0.0001 |
| *egl-9(sa307)* | − ^e^ | 14.9 | 103% | 89 | 0.3491 |
| N2 | control | 14.0 | − | 82 | − |
| N2 | *hif-1* | 16.3 | 116% | 78 | <0.0001 |
| *daf-16(mgDf47)* | control | 10.9 | − | 92 | − |
| *daf-16(mgDf47)* | *hif-1* | 12.5 | 115% | 79 | <0.0001 |
| *daf-2(e1370)* | control | 28.8 | − | 78 | − |
| *daf-2(e1370)* | *hif-1* | 35.5 | 123% | 99 | 0.0075 |
| *unc-24(e138) daf-15(m634)/nT1* | control | 16.3 | − | 94 | − |
| *unc-24(e138) daf-15(m634)/nT1* | *hif-1* | 15.8 | 97% | 99 | 0.1537 |
| N2 | − ^e^ | 13.3 | − | 99 | − |
| *hif-1(ia04)* | − ^e^ | 16.9 | 127% | 94 | <0.0001 |
| *egl-9(sa307)* | − ^e^ | 14.4 | 108% | 78 | 0.0393 |
| *rsks-1(ok1255)* | − ^e^ | 16.3 | 123% | 82 | <0.0001 |
| *rsks-1(ok1255); hif-1(ia04)* ^f^ | − ^e^ | 17.2 | 129% | 99 | <0.0001 |
| *rsks-1(ok1255); egl-9(sa307)* ^g^ | − ^e^ | 14.3 | 108% | 78 | 0.0356 |

^a^ average lifespan in days.

^b^ percentages were calculated using the mean lifespan.

^c^ numbers of animals scored.

^d^ *p*-values were calculated for log-rank tests.

^e^ animals were treated with *E. coli* OP50 food under standard lab conditions.

^f^ log-rank test: *rsks-1* vs. *rsks-1; hif-1*, *p* = 0.1147.

^g^ log-rank test: *rsks-1* vs. *rsks-1; egl-9*, *p* = 0.0015.
