# Supplementary material for: HIF-1 Modulates Dietary Restriction-Mediated Lifespan Extension via IRE-1 in Caenorhabditis elegans
Source: PLoS Genet. 2009 May 22;5(5):e1000486. doi: 10.1371/journal.pgen.1000486 (PMC2676694; doi:10.1371/journal.pgen.1000486)
Supplement: Table S2 — HIF-1 and IRE-1 mediate lifespan extension by DR. (0.06 MB DOC) [file pgen.1000486.s010.doc]

**Table S2. HIF-1 and IRE-1 mediate lifespan extension by DR**

| **Genotype** | **Food conc. (cfu / ml)** | **Mean lifespan ^a^** | **Percent of control ^b^** | **n ^c^** | ***p*-value vs. control ^d^** |
| --- | --- | --- | --- | --- | --- |
| N2 | 1.0 × 10^12^ | 12.7, 12.2 | − | 59, 54 | − |
| N2 | 1.0 × 10^11^ | 15.0, 14.6 | − | 58, 57 | − |
| N2 | 1.0 × 10^10^ | 18.7, 19.0 | − | 55, 60 | − |
| N2 | 1.0 × 10^9^ | 20.2, 23.0 | − | 57, 52 | − |
| N2 | 1.0 × 10^8^ | 19.0, 21.6 | − | 57, 34 | − |
| *hif-1(ia04)* | 1.0 × 10^12^ | 16.8, 16.0 | 132%, 131% | 53, 43 | <0.0001, <0.0001 |
| *hif-1(ia04)* | 1.0 × 10^11^ | 17.7, 17.1 | 108%, 117% | 47, 51 | <0.0001, <0.0001 |
| *hif-1(ia04)* | 1.0 × 10^10^ | 19.0, 21.1 | 112%, 111% | 38, 56 | 0.1916, <0.0001 |
| *hif-1(ia04)* | 1.0 × 10^9^ | 19.2, 23.0 | 95%, 100% | 52, 52 | 0.0935, 0.9370 |
| *hif-1(ia04)* | 1.0 × 10^8^ | 19.4, 22.9 | 102%, 106% | 57, 38 | 0.2367, 0.0683 |
| *egl-9(sa307)* | 1.0 × 10^12^ | 13.9, 12.6 | 109%, 103% | 34, 74 | 0.2214, 0.2110 |
| *egl-9(sa307)* | 1.0 × 10^11^ | 13.4, 14.6 | 89%, 100% | 30, 82 | 0.0001, 0.6195 |
| *egl-9(sa307)* | 1.0 × 10^10^ | 17.2, 17.4 | 92%, 92% | 59, 81 | 0.3917, <0.0001 |
| *egl-9(sa307)* | 1.0 × 10^9^ | 18.0, 17.8 | 89%, 77% | 50, 86 | <0.0001, <0.0001 |
| *egl-9(sa307)* | 1.0 × 10^8^ | 11.2, 14.2 | 59%, 66% | 48, 73 | <0.0001, <0.0001 |
| *ire-1(v33)* | 1.0 × 10^12^ | 9.8, 9.5 | 77%, 78% | 58, 58 | <0.0001, <0.0001 |
| *ire-1(v33)* | 1.0 × 10^11^ | 10.2, 9.7 | 68%, 66% | 59, 59 | <0.0001, <0.0001 |
| *ire-1(v33)* | 1.0 × 10^10^ | 10.2, 10.0 | 55%, 53% | 59, 60 | <0.0001, <0.0001 |
| *ire-1(v33)* | 1.0 × 10^9^ | 11.7, 11.9 | 58%, 52% | 58, 60 | <0.0001, <0.0001 |
| *ire-1(v33)* | 1.0 × 10^8^ | 11.1, 10.5 | 58%, 49% | 53, 55 | <0.0001, <0.0001 |
| N2 | − ^e^ | 15.2 | − | 120 | − |
| *egl-9(sa307)* | − ^e^ | 15.2 | 100% | 68 | 0.4060 |
| *eat-2(ad1116)* | − ^e^ | 18.2 | 120% | 98 | <0.0001 |
| *eat-2(ad1116);egl-9(sa307)* ^f^ | − ^e^ | 15.5 | 102% | 95 | 0.4448 |

^a^ average lifespan in days.

^b^ changes in mean lifespan compared to N2 growing at the same food concentration.

^c^ numbers of animals scored.

^d^ *p*-values were calculated for log-rank tests by comparison to N2 growing at the same food concentration.

^e^ animals were treated with *E. coli* OP50 food under standard lab conditions.

^f^ log-rank tests: *eat-2* vs. *eat-2; egl-9*, *p* < 0.0001.
