# Supplementary material for: HIF-1 Modulates Dietary Restriction-Mediated Lifespan Extension via IRE-1 in Caenorhabditis elegans
Source: PLoS Genet. 2009 May 22;5(5):e1000486. doi: 10.1371/journal.pgen.1000486 (PMC2676694; doi:10.1371/journal.pgen.1000486)
Supplement: Table S3 — HIF-1 functions in specific neurons and muscles to modulate DR-dependent lifespan extension. (0.04 MB DOC) [file pgen.1000486.s011.doc]

**Table S3. HIF-1 functions in specific neurons and muscles to modulate DR-dependent lifespan extension**

| **Strain** | **Genotype** | **Mean lifespan ^a^** | **Percent of control ^b^** | **n ^c^** | ***p*-value vs. N2 ^d^** | ***p*-value vs. JT307 ^e^** |
| --- | --- | --- | --- | --- | --- | --- |
| N2 |  | 20.6 | − | 59 | − | <0.0001 |
| JT307 | *egl-9(sa307)* | 16.7 | 81% | 60 | <0.0001 | − |
| CX8756 | *egl-9 (sa307); kyEx1593 [egl-9::egl-9::gfp]* | 21.4 | 104% | 47 | 0.0657 | <0.0001 |
| CX10149 | *egl-9 (sa307); kyEx2321 [H20::egl-9::gfp, tdc-1::egl-9::gfp]* | 20.2 | 98% | 57 | 0.8673 | <0.0001 |
| CX8628 | *egl-9 (sa307); kyEx1525 [H20::egl-9::gfp]* | 19.4 | 94% | 57 | 0.0694 | <0.0001 |
| CX10090 | *egl-9 (sa307); kyEx2288 [tdc-1::egl-9::gfp]* | 17.8 | 96% | 53 | <0.0001 | 0.1963 |
| CX8632 | *egl-9 (sa307); kyEx1529 [tph-1::egl-9::gfp]* | 20.6 | 100% | 59 | 0.8701 | <0.0001 |
| CX8832 | *egl-9 (sa307); kyEx1639 [gcy-36::egl-9::gfp]* | 17.4 | 91% | 48 | <0.0001 | 0.2358 |
| CX8630 | *egl-9 (sa307); kyEx1527 [myo-3::egl-9::gfp]* | 20.5 | 100% | 56 | 0.4368 | <0.0001 |
| CX9889 | *egl-9 (sa307); kyEx2215 [hum-5::egl-9::gfp]* | 13.7 | 67% | 53 | <0.0001 | <0.0001 |
| CX8792 | *egl-9 (sa307); kyEx1616 [myo-2::egl-9::gfp]* | 19.2 | 93% | 57 | 0.2308 | <0.0001 |

^a^ average lifespan in days.

^b^ changes in mean lifespan compared to N2.

^c^ numbers of animals scored.

^d^ *p*-values were calculated for log-rank tests by comparison to N2.

^e^ *p*-values were calculated for log-rank tests by comparison to JT307 *egl-9(sa307)*.
