# Supplementary material for: HIF-1 Modulates Dietary Restriction-Mediated Lifespan Extension via IRE-1 in Caenorhabditis elegans
Source: PLoS Genet. 2009 May 22;5(5):e1000486. doi: 10.1371/journal.pgen.1000486 (PMC2676694; doi:10.1371/journal.pgen.1000486)
Supplement: Table S4 — Tissue-specific rescue of egl-9 does not affect lifespan under AL. (0.04 MB DOC) [file pgen.1000486.s012.doc]

**Table S4. Tissue-specific rescue of *egl-9*** does not affect lifespan under AL

| **Strain** | **Genotype** | **Mean lifespan ^a^** | **Percent of control ^b^** | **n ^c^** | ***p*-value vs. N2 ^d^** | ***p*-value vs. JT307 ^e^** |
| --- | --- | --- | --- | --- | --- | --- |
| N2 |  | 15.5 | − | 59 | − | 0.0497 |
| JT307 | *egl-9(sa307)* | 14.3 | 92% | 50 | 0.0497 | − |
| CX8756 | *egl-9 (sa307); kyEx1593 [egl-9::egl-9::gfp]* | 14.0 | 90% | 43 | 0.0063 | 0.4426 |
| CX10149 | *egl-9 (sa307); kyEx2321 [H20::egl-9::gfp, tdc-1::egl-9::gfp]* | 16.0 | 103% | 55 | 0.1302 | 0.0024 |
| CX8628 | *egl-9 (sa307); kyEx1525 [H20::egl-9::gfp]* | 15.3 | 99% | 60 | 0.8119 | 0.0522 |
| CX10090 | *egl-9 (sa307); kyEx2288 [tdc-1::egl-9::gfp]* | 14.8 | 95% | 49 | 0.4633 | 0.2531 |
| CX8632 | *egl-9 (sa307); kyEx1529 [tph-1::egl-9::gfp]* | 15.4 | 99% | 54 | 0.6443 | 0.0325 |
| CX8832 | *egl-9 (sa307); kyEx1639 [gcy-36::egl-9::gfp]* | 14.3 | 92% | 46 | 0.0432 | 0.9378 |
| CX8630 | *egl-9 (sa307); kyEx1527 [myo-3::egl-9::gfp]* | 15.2 | 98% | 42 | 0.8247 | 0.0789 |
| CX9889 | *egl-9 (sa307); kyEx2215 [hum-5::egl-9::gfp]* | 14.9 | 96% | 44 | 0.6813 | 0.2213 |
| CX8792 | *egl-9 (sa307); kyEx1616 [myo-2::egl-9::gfp]* | 13.8 | 85% | 38 | 0.0067 | 0.3943 |

Lifespan of N2, JT307 *egl-9(sa307)* and *egl-9* animals with various tissue-specific promoters driving *egl-9* cDNA (CX strains) was measured under AL.

^a^ average lifespan in days.

^b^ changes in mean lifespan compared to N2.

^c^ numbers of animals scored.

^d^ *p*-values were calculated for log-rank tests by comparison to N2.

^d^ *p*-values were calculated for log-rank tests by comparison to JT307 *egl-9(sa307)*.
