# Supplementary material for: HIF-1 Modulates Dietary Restriction-Mediated Lifespan Extension via IRE-1 in Caenorhabditis elegans
Source: PLoS Genet. 2009 May 22;5(5):e1000486. doi: 10.1371/journal.pgen.1000486 (PMC2676694; doi:10.1371/journal.pgen.1000486)
Supplement: Table S5 — IRE-1 is required for lifespan extension by hif-1. (0.03 MB DOC) [file pgen.1000486.s013.doc]

**Table S5. IRE-1 is required for lifespan extension by *hif-1***

| **Genotype** | **Food conc. (cfu / ml)** | **Mean lifespan ^a^** | **Percent of control ^b^** | **n ^c^** | ***p*-value vs. control ^d^** |
| --- | --- | --- | --- | --- | --- |
| N2 | 1.0 × 10^11^ | 15.0 | − | 58 | − |
| N2 | 1.0 × 10^9^ | 20.2 | − | 57 | − |
| *ire-1(v33)* | 1.0 × 10^11^ | 10.2 | 68% | 59 | <0.0001 |
| *ire-1(v33)* | 1.0 × 10^9^ | 11.7 | 58% | 58 | <0.0001 |
| *hif-1(ia04)* | 1.0 × 10^11^ | 17.7 | 118% | 47 | <0.0001 |
| *hif-1(ia04)* | 1.0 × 10^9^ | 19.2 | 95% | 52 | 0.0935 |
| *ire-1(v33); hif-1(ia04)* ^e^ | 1.0 × 10^11^ | 8.8 | 59% | 58 | 0.0012 |
| *ire-1(v33); hif-1(ia04)* ^f^ | 1.0 × 10^9^ | 11.9 | 59% | 60 | 0.8406 |

^a^ average lifespan in days.

^b^ changes in mean lifespan compared to N2 growing at the same food concentration.

^c^ numbers of animals scored.

^d^ *p*-values were calculated for log-rank tests by comparison to N2 growing at the same food concentration.

^e^ log-rank test: *ire-1* AL vs. *ire-1; hif-1* AL, *p* = 0.9076.

^f^ log-rank test: *ire-1* DR vs. *ire-1; hif-1* DR, *p* = 0.5445.
